# A single-cell transcriptomic atlas of the periventricular proliferative zone in the late gestation fetal brain in the pigtail macaque

**DOI:** 10.3389/fnins.2026.1785001

**Published:** 2026-08-03

**Authors:** John A. Cornelius, Megana Shivakumar, Taeyun Kim, Orlando Cervantes, Raj P. Kapur, Hazel Huang, Hong Zhao, Briana M. Del Rosario, Amanda Li, Richard J. Li, Sidney Sun, Andrew E. Vo, Gygeria Manuel, Akshaj Bharadwaj, Jeff Munson, Miranda Li, Edmunda Li, Sophia Chima, Austyn Orvis, Michelle Coleman, Alexandra Christodolou, Melissa R. Berg, Britni C. Curtis, Brenna A. Menz, Jin Dai, Inah D. Golez, Solomon N. Wangari, Stephen A. McCartney, Kristen Noble, Pilar Flores-Espinoza, Addy Cecilia Helguera-Repetto, Andrea Olmos-Ortiz, Monica Selena Fonseca-Perez, Chris English, Audrey Baldessari, Nardhy Gomez-Lopez, Rebecca Hodge, Lakshmi Rajagopal, Kristina M. Adams Waldorf

**Affiliations:** 1 Department of Obstetrics and Gynecology, University of Washington, Seattle, WA, United States; 2 Pacific Northwest University of Health Sciences (PNWU) College of Osteopathic Medicine, Yakima, WA, United States; 3 Department of Global Health, University of Washington, Seattle, WA, United States; 4 Department of Laboratory Medicine and Pathology, University of Washington, Seattle, WA, United States; 5 Seattle Children's Hospital, Seattle, WA, United States; 6 Case Western Reserve University School of Medicine, Cleveland, OH, United States; 7 Columbia University, New York, NY, United States; 8 University of Washington School of Medicine, Seattle, WA, United States; 9 Morehouse School of Medicine, Atlanta, GA, United States; 10 Department of Psychiatry, University of Washington, Seattle, WA, United States; 11 Center for Global Infectious Disease Research, Seattle Children's Research Institute, Seattle, WA, United States; 12 Washington National Biomedical Research Center, Seattle, WA, United States; 13 Department of Pediatrics, Vanderbilt University Medical Center, Nashville, TN, United States; 14 Department of Obstetrics and Gynecology, University of Texas Medical Branch Galveston, Galveston, TX, United States; 15 Department of Immunobiochemistry, National Institute of Perinatology, Mexico City, Mexico; 16 Departments of Obstetrics and Gynecology and Pathology and Immunology, Center for Reproductive Health Sciences, Washington University in St. Louis, St. Louis, MO, United States; 17 Allen Brain Institute, Seattle, WA, United States

**Keywords:** astrocyte, brain, fetus, neuron, microglia, neurodevelopment, neuroprogenitor, nonhuman primate

## Abstract

**Background:**

The fetal brain undergoes rapid cellular and structural changes in late gestation, when waves of neurogenesis and gliogenesis shape cortical circuitry. The ventricular zone (VZ), subventricular zone (SVZ), periventricular white matter (PVWM), and deep white matter (DWM) are enriched in neuroprogenitor cells, newborn neurons, and interneurons, which are regions challenging to study in the third-trimester human fetal brain. The nonhuman primate (NHP) provides a powerful translational model to overcome this limitation, given its close similarity to human neurodevelopmental trajectories. The study objective was to construct a single-cell RNA-Seq (scRNA-Seq) atlas of the late-gestation fetal brain of the pigtail macaque (*Macaca nemestrina*), focusing on cells in the periventricular proliferative zone.

**Methods:**

A sample of the lateral ventricular wall, subventricular zone, and overlying white/gray matter was dissociated into single cells and processed through 10X Genomics sequencing, SoupX removal of ambient RNA, empty droplet removal, doublet exclusion, and Seurat's pipeline which consists of aggregation, unsupervised clustering, and cluster annotation to create a single-cell RNA-Seq (scRNA-Seq) atlas. We also investigated developmental trajectories using scVelo and CellRank2.

**Results:**

This analysis captured diverse populations of neuroprogenitors, newborn neurons, developing lineages of excitatory and inhibitory neurons, oligodendrocytes, astrocytes, epithelial and vascular cells.

**Conclusions:**

Single-cell populations from the third-trimester nonhuman primate fetal brain are highly similar to those in the human fetus at the level of major lineages. This late-gestation single-cell atlas of the periventricular proliferative zone provides a unique reference for progenitor, neuronal, glial, vascular, and immune cell states during a critical window of primate neurodevelopment, enabling mechanistic interrogation of how inflammatory, infectious, or hypoxic insults disrupt vulnerable neurogenic niches.

## Background

The fetal brain is a dynamic organ that undergoes rapid cellular proliferation, differentiation, and migration to establish complex cortical circuitry and complex neurologic functions. In the developing fetal brain, the ventricular zone (VZ), subventricular zone (SVZ), periventricular white matter (PVWM), and deep white matter (DWM) represent critical regions enriched in neuroprogenitor cells and newborn neurons. These neurogenic niches are highly vulnerable to injury from pathogens and inflammatory insults that access the cerebrospinal fluid (CSF), such as Zika virus (ZIKV), cytomegalovirus (CMV), and diverse bacterial infections. In addition to congenital infections, the periventricular proliferative zone is highly sensitive to sterile inflammatory exposures, hypoxia-ischemia, and preterm birth-associated immune activation, all of which converge on these neurogenic niches during late gestation. Experimental models, such as nonhuman primates (NHPs), are required to study these events because normal human fetal brain tissue at this late gestational stage is rarely accessible for ethical reasons.

NHPs provide a powerful model for human neurodevelopment due to similarities in gestational length, cortical structure, and timing of neurogenesis. Among the most used species are rhesus macaques (*Macaca mulatta*), pigtail macaques (*Macaca nemestrina*), and cynomolgus macaques (*Macaca fascicularis*), which have been utilized to study viral neuropathogenesis (Adams Waldorf et al., 2018, 2016; Kapur et al., 2025; Barry et al., 2006), neuroprogenitor and white matter injury (Kapur et al., 2025; Short et al., 2010; Martinot et al., 2018; Saron et al., 2023), and neurodevelopmental outcomes after *in utero* exposures (Moadab et al., 2023). Although NHP models are widely employed to study how experimental insults affect the developing brain, the extent to which they recapitulate the diversity of human neural progenitors, glial precursors, and newborn neurons has been incompletely defined. While there are differences in the species-specific rates of neurogenesis and gliogenesis, the structure and organization of the late-gestation macaque brain closely resemble those of the human brain; there is a similar laminar organization of the SVZ and subplate, and parallel timelines of oligodendrocyte maturation and axonal growth (Malkova et al., 2006; Liu et al., 2020; Wang et al., 2015; Smart et al., 2002; Martínez-Cerdeño et al., 2012). NHPs are one of the most translationally relevant animal models for investigating human fetal neurodevelopment and its susceptibility to infection and injury.

Single-cell RNA sequencing (scRNA-Seq) has transformed the study of complex tissues by enabling detailed investigation of cellular heterogeneity and transcriptional programs. Several scRNA-Seq atlases of the human brain have been generated, primarily from mid-gestation samples (gestational weeks 15–24), providing critical insights into progenitor populations, lineage trajectories, and neuronal subtype specification (Aldinger et al., 2021). However, human datasets from late gestation are extremely limited due to tissue availability. In contrast, scRNA-Seq studies of the NHP fetal brain are rare, with only a handful of reports describing bulk or spatial transcriptomics, organoids, or specific progenitor cell populations (Adams Waldorf et al., 2016, 2018; Tisoncik-Go et al., 2024).

Our objective was to construct a scRNA-Seq atlas of the late gestation pigtail macaque fetal brain, focusing on cell populations in the periventricular proliferative zone and the adjacent white and gray matter, which are vulnerable to infection or inflammatory injury due to exposure to cerebrospinal fluid. By characterizing progenitor, neuronal, glial, and immune cell populations at single-cell resolution, we provide a reference dataset for the normal development of these vulnerable regions. This atlas also establishes a foundation for comparative studies with human fetal brain datasets and provides a resource for investigating how congenital infections or inflammatory exposures alter neurodevelopmental trajectories.

## Results

### Study design

To determine the single-cell transcriptional profiles of the fetal pigtail macaque within the periventricular proliferative zone and adjacent white/gray matter, we collected tissues from these regions for single-cell dissociation and analysis using the 10X Genomics pipeline. Tissues were obtained from 9 healthy, uninfected fetal pigtail macaques, serving as historical controls for infectious disease studies. They had either undergone surgical catheterization and saline inoculation of the amniotic cavity and choriodecidual space (*N* = 4) or subcutaneous media inoculation of the forearm (*N* = 5; Table 1). All animals were delivered by Cesarean section, and none developed preterm labor or other perinatal complications. Fetal brain tissues from the periventricular zone and adjacent white/gray matter were single-cell dissociated and processed through the 10X Genomics scRNA-Seq pipeline and integrated using the Seurat analysis pipeline. Cell counts by animal are shown in Supplementary Table S1.

**Table 1:** Animal demographics.

Animal ID	Inoculum	Maternal age at delivery (years)	Gestational age at inoculation (days)	Gestational age at delivery (days)	Fetal body weight (g)	Fetal brain weight (g)	Fetal sex
CTRL1	Catheterized	11.3	134.4	135.4	337.6	46.2	F
CTRL2	Catheterized	5.7	127.3	128.4	242.0	UNK	M
CTRL3	Catheterized	6.1	133.4	134.4	264.0	40.1	F
CTRL4	Catheterized	12.7	131.3	132.3	298.6	42.8	F
CTRL5	Media	5.2	138.0	158.4	499.2	55.9	F
CTRL6	Media	10.8	127.4	147.4	469.0	61.3	M
CTRL7	Media	12.9	141.3	144.3	412.6	57.7	M
CTRL8	Media	16.3	154.0	157.4	441.9	59.4	F
CTRL9	Media	5.3	145.4	148.4	340.3	49.5	F

This table shows the inoculum, maternal age at delivery, gestational age at inoculation and delivery, fetal body weight, fetal brain weight, and fetal sex for each animal in the study. Abbreviations: F, female; g, grams; M, male; UNK, unknown.

### Comparative anatomy of the late gestation fetal pigtail macaque and early infant human fetal SVZ and deep white matter

Creation of a scRNA-Seq atlas of the fetal pigtail macaque SVZ and deep white matter necessitates an understanding of anatomical similarities and differences with human fetuses at the same time point (Figure 1). As normal third-trimester human fetal brains are rarely obtained and described, we compared the same anatomical structures with an early infant brain. Overall, the architecture of the fetal SVZ and deep white matter is remarkably similar between the two species.

**Figure 1:**
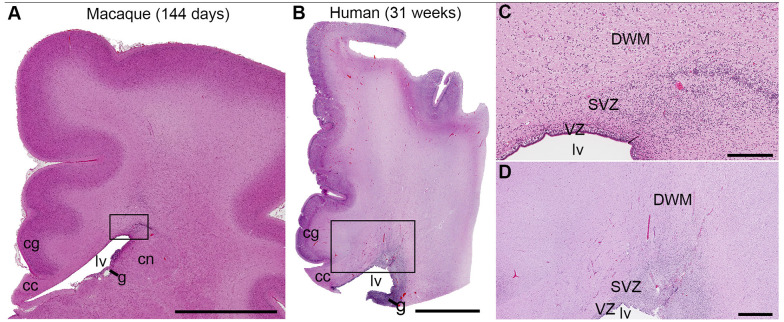
Cerebral cortical histology of a pigtail macaque and human third-trimester fetuses at comparable ages. This figure shows images of the cerebral cortex from pigtail macaque and human fetuses at comparable gestational ages in the third trimester (macaque: 144 days, term gestation: 172 days; human: 31 weeks, term gestation 40 weeks). **(A)** A low magnification image of a hematoxylin-and-eosin-stained coronal section through the dorsal portion of a macaque cerebral hemisphere includes the lateral ventricle (lv) with adjacent ganglionic eminence (g), cortical gray matter (cg), corpus callosum (cc) and caudate nucleus (cn). **(B)** The medial half of a similar section from a comparable stage human fetus shows many of the same landmarks, although the human brain is significantly larger. **(C)** Higher magnification of the rectangular region outlined in **(A)** illustrates relative positions of the ventricular zone (VZ), subventricular zone (SVZ), and deep white matter (DWM). **(D)** Higher magnification of the rectangular region outlined in **(B)** shows the locations of the same zones in the human fetus. Scale bars: **(A)** 5 mm; **(B)** 5 mm; **(C)** 300 μm; **(D)** 1 mm.

### Clustering, annotation, and cell origin

To characterize the cellular composition of the developing fetal brain, we performed droplet-based single-cell RNA sequencing across nine samples, recovering 35,836 high-quality cells after ambient RNA correction (SoupX), empty droplet removal (emptyDrops), doublet exclusion (scDblFinder), and filtering on gene complexity and mitochondrial content. Sample-driven batch effects were corrected using Harmony integration, yielding a unified embedding (Figure 2). Unsupervised clustering identified 27 transcriptionally distinct populations, which were manually annotated using established lineage-specific marker genes, comparative primate and human fetal brain datasets, and trajectory relationships (Figure 3A). Differential abundance analyses between the catheterized group (CTRL1–4) and the media group (CTRL5–9) did not show significant differences in the proportion of any single cell type between the groups (Supplementary Figure S1). We also did not observe significant differences in proportion of any single cell type by fetal sex (Supplementary Figure S2).

**Figure 2:**
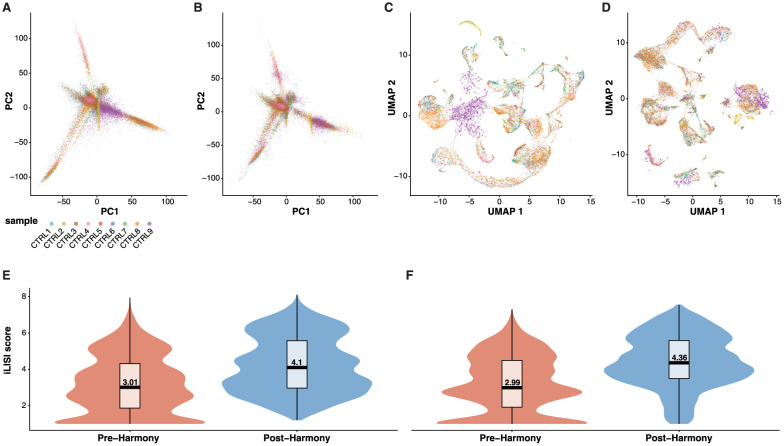
Quality control analyses of cell populations. **(A–D)** PCA and UMAP embeddings of single-cell populations colored by sample identity before **(A, C)**, and after **(B, D)** Harmony batch correction, demonstrating the effect of integration across catheterized (CTRL1-4) and media inoculation (CTRL5-9) control samples. **(E, F)** Violin plots displaying the integration Local Inverse Simpson's Index (iLISI) score before and after Harmony batch correction, with median values shown. Higher iLISI scores indicate greater sample mixing and improved batch correction. Each dot represents an individual cell.

**Figure 3:**
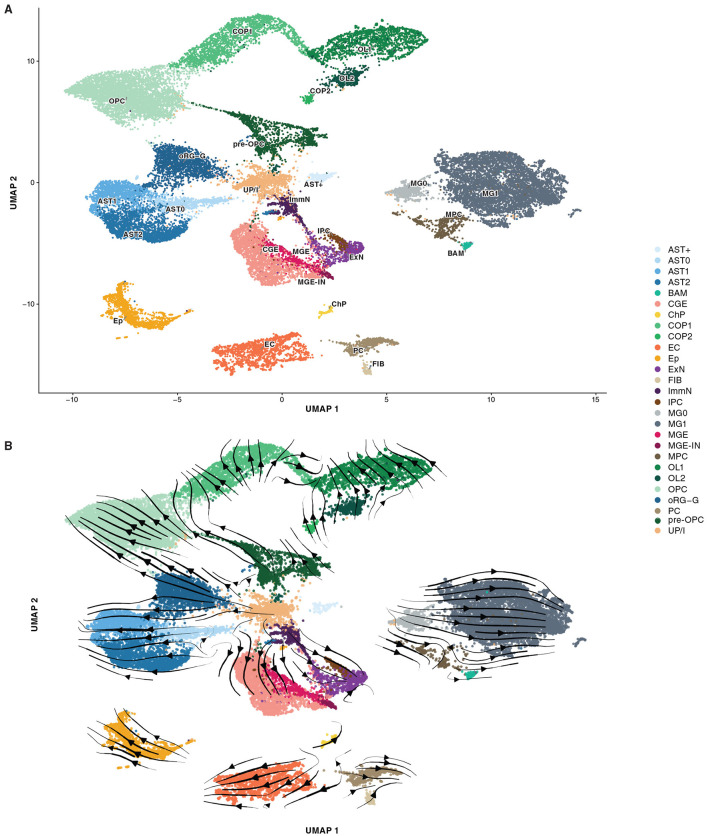
Single-cell atlas of the periventricular proliferative region of the late gestation pigtail macaque fetal brain. **(A)** UMAP of single-cell transcriptomes illustrates distinct cellular clusters identified by unsupervised clustering. Each point represents an individual cell, colored according to cluster. **(B)** RNA velocity trajectories overlaid on the UMAP embedding, with arrows indicating the predicted direction of differentiation for each cell population. AST, astrocyte; BAM, border-associated macrophage; CGE, caudal ganglionic eminence lineage; ChP, choroid plexus; COP, committed oligodendrocyte precursor; EC, endothelial cell; Ep, ependymal cell; ExN, excitatory neuron lineage; FIB, fibroblast; ImmN, immature neurons; IPC, intermediate progenitor cell; MG, microglia; MGE, medial ganglionic eminence lineage; MGE-IN, MGE-derived interneuron; MPC, mesenchymal progenitor cell; OL, oligodendrocyte; OPC, oligodendrocyte progenitor cell; oRG-G, outer radial glia undergoing gliogenic differentiation; PC, pericyte; pre-OPC, pre-oligodendrocyte progenitor cell; UP/I, undifferentiated progenitor cell/intermediate state.

Cell populations were visualized using a uniform manifold approximation and projection (UMAP) plot, and then trajectory analysis was performed using scVelo (Figure 3B). An undifferentiated progenitor/intermediate (UP/I) population was at the center of the UMAP with most populations radiating outward from (or inward to) the UP/I. Key populations near the UP/I were annotated as pre-oligodendrocyte progenitor cell (pre-OPC), outer radial glia undergoing gliogenic differentiation (oRG-G), immature neurons (ImmN), immature astrocytes (AST0), pericytes (PC), and a population of committed oligodendrocyte precursors (COP2). The cell cycle status was also evaluated using Seurat's CellCycleScoring (Supplementary Figure S3). We found a heterogeneous mitotic state in the UP/I, and a highly mitotic state in pre-OPC and oRG-G, consistent with committed progenitors.

### Neuronal lineages

Several populations of developing neurons were identified including caudal ganglionic eminence cells (CGE), excitatory neurons (ExN), ImmN, intermediate progenitor cells (IPC), medial ganglionic eminence cells (MGE) and MGE-derived interneurons (MGE-IN; Figure 4). IPCs arising from radial glia in the periventricular proliferative zone undergo several rounds of cell division before differentiating into glutamatergic projection neurons that populate both the deep layers (DL, layers V-VI) and upper layers (UL, layers II-IV) of the neocortex. The IPC population in the periventricular proliferative zone expressed *EOMES* (*TBR2*) and *TBR1*, consistent with a late transitional IPC state that exited the cell cycle and is transcriptionally committed to a deep-layer neuronal fate (Figures 4B–D). UMAP feature plots illustrated a progressive maturation from *EOMES*^+^ early neurons (IPCs) toward *SATB*2^+^ differentiated populations (ExN). RNA expression within the ExN population was consistent with a transitional subplate neuronal state that had initiated upper-layer and callosal projection neuron transcriptional programs (*CUX2, SATB2*) while retaining residual deep-layer markers (*TBR1*; Figures 4B,C). ExN also expressed *SLC17A6* (*VGLUT2*), a canonical marker of glutamatergic neurons that is highly expressed in early born deep-layer excitatory neurons and subcortical projection neurons (Figure 4B). In contrast, the ImmN population expressed *SLC17A7* (*VGLUT1*), *NRGN*, and *CAMK2A*, but few other markers of mature excitatory or inhibitory neurons, suggesting an immature state (Figure 4B). Trajectory analyses indicated that ExN was derived from both ImmN and IPCs (Supplementary Figure S4).

**Figure 4:**
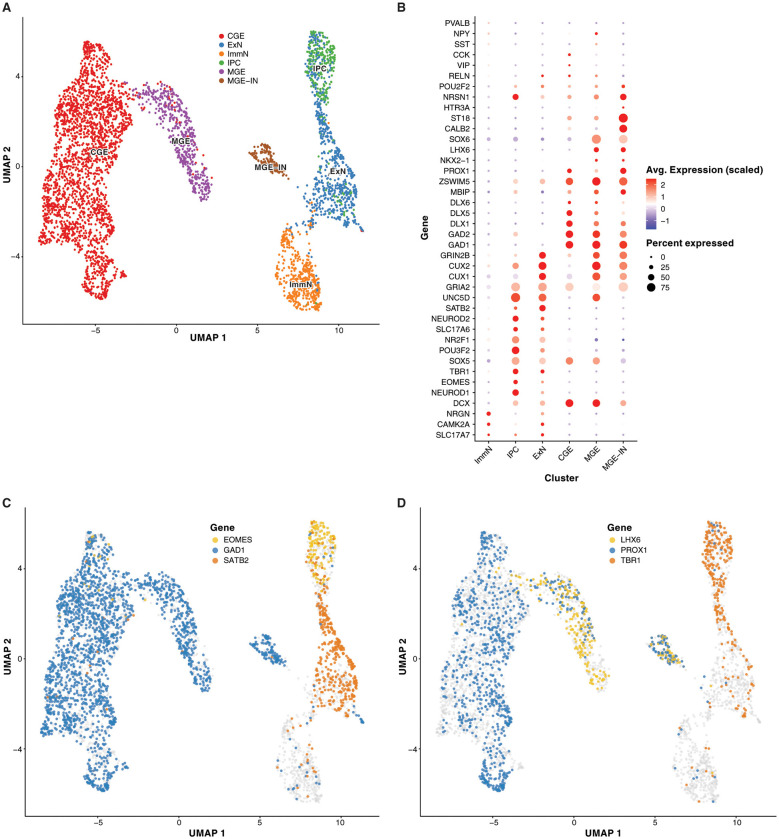
Transcriptomic profile of neuronal lineage cells. **(A)** UMAP visualization of neuron populations identified by single-cell RNA-seq. Each point represents an individual cell, and colors denote cluster identity. **(B)** Dot plot summarizing the expression of representative neuronal marker genes across clusters. Dot size reflects the proportion of cells expressing each gene, while color intensity indicates scaled average expression. **(C)** UMAP feature plot showing expression of key transcriptional markers *EOMES* (yellow), *GAD1* (blue), and *SATB2* (orange), highlighting excitatory and inhibitory neuronal lineages. **(D)** UMAP feature plot displaying expression of *LHX6* (yellow), *PROX1* (blue), and *TBR1* (orange), defining marker genes for MGE, CGE, and excitatory neuron lineages, respectively.

Medial and caudal ganglionic eminences (MGE and CGE, respectively) produce GABAergic interneurons in the developing fetal NHP and human brain. These eminences form proliferative zones within the ventral telencephalon, located along the ventral and medial walls of the lateral ventricles. The CGE extends caudally and produces *VIP*^+^, *CALB*2^+^, and *NR*2*F*2^+^ interneurons that migrate into superficial cortical layers. Together, the MGE and CGE transient germinal structures generate most GABAergic inhibitory interneurons in the brain. MGE-IN was defined by *LHX6, GAD1, GAD2, CALB2*, and *ST18* expression (Figures 4B,D). Although the MGE gives rise to *PVALB*^+^ and *SST*^+^ interneurons and basal ganglia projection neurons, *PVALB* was not expressed in these clusters, indicating that these MGE interneurons were relatively immature. A population of CGE-derived progenitors, labeled CGE, was characterized by the expression of *PROX1, DLX1, DLX5, GAD2*, and weak expression of *RELN, VIP*, and *CCK* (Figure 4D). Expression of GABAergic markers in both the MGE and CGE lineage (*GAD1, GAD2*, and *DLX2*) reflected their inhibitory identity. In summary, these data delineate transcriptionally distinct progenitor and interneuron precursor populations in the developing ventral forebrain adjacent to the subventricular zone.

### Oligodendrocyte lineage

One progenitor and five cell populations within the oligodendrocyte lineage were identified by expression of canonical markers, including *SOX10, PDGFRA, PCDH17, OLIG2, BCAS1, MAL, PLP1*, and *MBP* (Figure 5). A pre-OPC population was identified as committed to the oligodendrocyte lineage high expression of cell cycle genes, including *MKI67*; this population also had nascent expression of *NKX2-2* (ENSMMUG00000056728), indicating oligodendrocyte commitment, alongside low expression of oligodendrocyte genes (i.e., *PDGFRA, PCDH15, MMP16*; Figure 5B). Of the oligodendrocyte lineage markers, pre-OPCs and OPCs expressed only *PDGFRA*, indicating a population entering the oligodendrocyte program (Figure 5C). Committed oligodendrocyte precursors (COPs) were defined by expression of *BCAS1*, indicating a transition from OPC to an early pre-myelinating or differentiating oligodendrocyte (Figure 5C). OL1 and OL2 expressed genes consistent with mature myelinating oligodendrocytes, including *MOG, PLP1, MBP*, and *MAL* (Figures 5B,C). The trajectory analysis revealed a progressive change in cellular maturity (ratio of spliced/unspliced RNA) from pre-OPC to OPC to COP1 cells (Figure 5D). Together, these analyses delineate the transcriptional progression from PDGFRA^+^ oligodendrocyte progenitors through *ENPP*6^+^ committed precursors to *MBP*^+^/*PLP*1^+^ mature oligodendrocytes.

**Figure 5:**
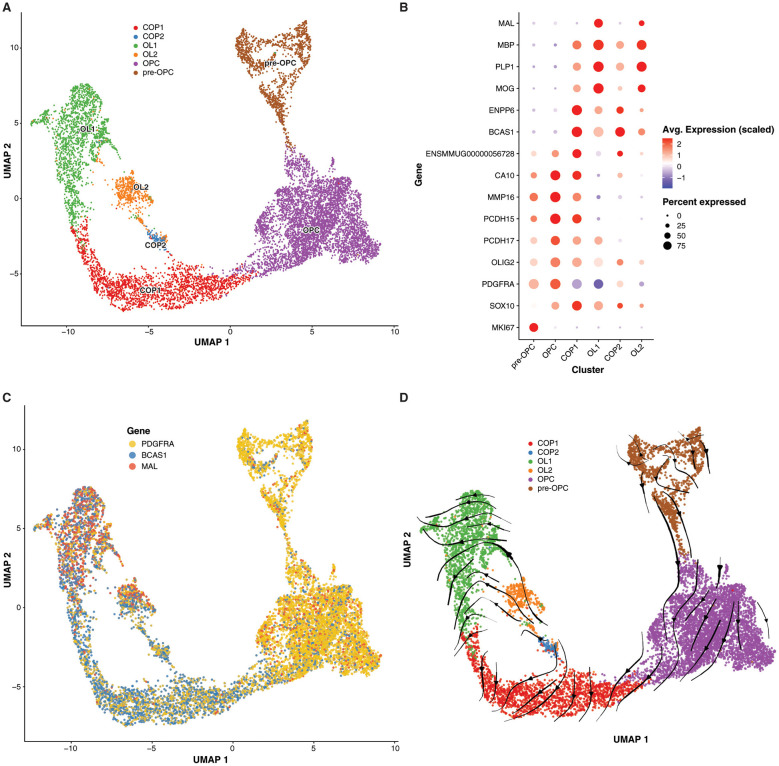
Transcriptomic profile of oligodendrocyte lineage cells. **(A)** UMAP visualization of oligodendrocyte lineage clusters identified by single-cell RNA-seq, including pre-oligodendrocyte progenitor cells (pre-OPC), oligodendrocyte progenitor cells (OPC), committed oligodendrocyte precursors (COP1–COP2), and maturing oligodendrocytes (OL1–OL2). Each point represents a single cell, and colors denote cluster identity, illustrating the continuum from progenitor to mature oligodendrocyte states. **(B)** Dot plot summarizing gene expression of key oligodendrocyte lineage marker genes across clusters. Dot size indicates the percentage of cells expressing each gene, and color intensity represents scaled average expression levels. **(C)** UMAP feature plot showing expression of *PDGFRA* (yellow), *BCAS1* (blue), and *MAL* (red), highlighting the transition from progenitor to mature oligodendrocyte states. **(D)** RNA velocity trajectories overlaid on the UMAP embedding, with arrows indicating the predicted direction of differentiation along the oligodendrocyte lineage.

### Astrocyte lineage

Astroglial populations clustered into four populations with one progenitor population (Figure 6A). A mitotically active oRG-G population expressed *EGFR, ASCL1, DLL1*, and *CDK6* with a trajectory into the astrocyte lineage (Figures 6B,D). AST0 gave rise to a reactive astrocyte population (AST+) expressing *HIF1A* (transcription factor for hypoxia response), *MT2A* (oxidative stress, inflammation), and *S100A6* with a concomitant drop in expression of *MT3*. AST0 had the greatest expression of *FABP7*, a canonical marker of immature astrocytes (Figure 6B). The AST1 population is a mature astrocyte population marked by the highest expression of *GLUL*, encoding the glutamine synthetase, and glutamate solute carriers (*SLC1A2, SLC1A3*). The expression profile of AST2 was most consistent with white matter fibrous or perivascular astrocytes, which constitutively express *GFAP, AQP4*, and *ID3* (Figures 6B,C). All astrocyte populations expressed *HOPX*, which is characteristic of interlaminar astrocytes descended from oRG-G in human and nonhuman primates (Figure 6C). Together, these data reveal transcriptionally distinct astrocyte subtypes that vary in expression of glutamate recycling machinery but retain expression of key markers (*HOPX*) that define primate interlaminar astrocytes.

**Figure 6:**
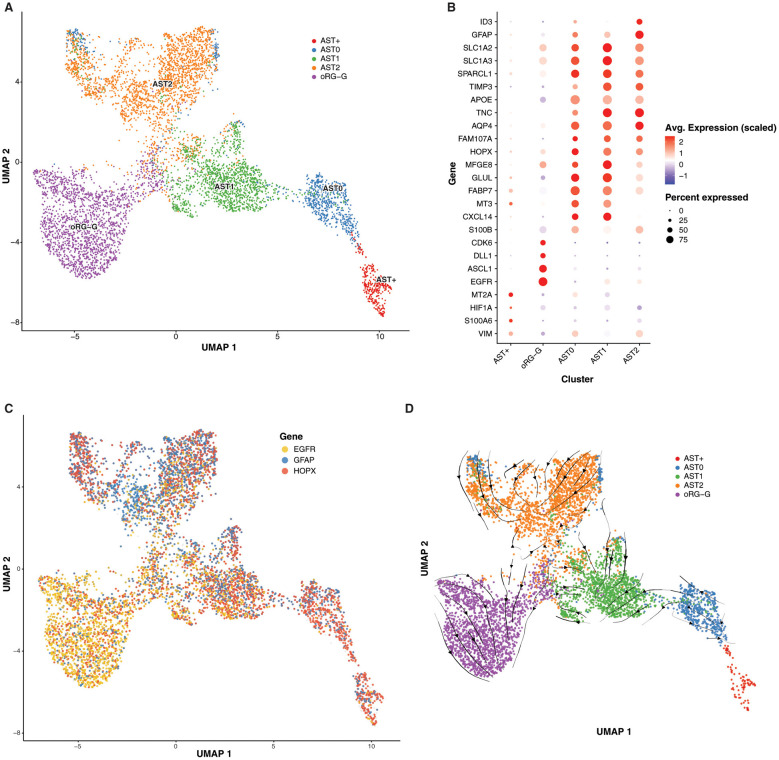
Transcriptomic profile of astrocyte lineage cells. **(A)** UMAP visualization of astrocyte clusters (AST+, AST0, AST1, AST2) and outer radial glia undergoing gliogenic differentiation (oRG-G) identified by single-cell RNA-seq. Each point represents an individual cell, and colors denote distinct subpopulations, illustrating transcriptional heterogeneity among astroglial cells. **(B)** Dot plot summarizing expression of representative astrocyte-enriched marker genes across clusters. Dot size indicates the percentage of cells expressing each gene, and color intensity represents scaled average expression levels. **(C)** UMAP feature plot showing expression of *EGFR* (yellow), *GFAP* (blue), and *HOPX* (red), highlighting key markers that distinguish astrocyte progenitor and mature astrocyte states. **(D)** RNA velocity trajectories overlaid on the UMAP embedding, with arrows indicating the predicted direction of differentiation along the astrocyte lineage.

### Microglia

Two populations of parenchymal microglia (MG0, MG1) and one border-associated microglia (BAM) were identified (Figure 7A). The parenchymal microglia expressed *AIF1* (IBA1), *CSF1R, TREM2, P2RY12*, and *CX3CR1* and denoting a homeostatic and surveillant phenotype (Figure 7B). BAM showed high expression of *CD163, LYVE1*, and *MRC1* with low to absent *SALL1* and *P2RY12*, a profile consistent with macrophages residing in the choroid plexus or ventricular surfaces (Figure 7B). No single animal or sample dominated the clustering of microglia (Supplementary Figure S5). Expression of canonical homeostatic and tissue residency markers was similar between MG0 and MG1 with greater expression of *MRC1, SALL1*, and *MERTK* in MG1 (Figures 7B, C, Supplementary Figure S6). Several markers of inflammatory activation and interferon response were more expressed in MG1 than MG0, including *CD80, CD86, IL1B, CLEC12A*, and *ITGAM* (Figure 7B, Supplementary Figure S7). To determine if MG1 and MG0 differed in M1-like markers, we analyzed *IL1B* and *CD86* as a function of pseudotime. We found that *IL1B* was a greater driver of MG1 pseudotime, particularly in the latest stages of differentiation (Supplementary Figure S8, Figure 7D).

**Figure 7:**
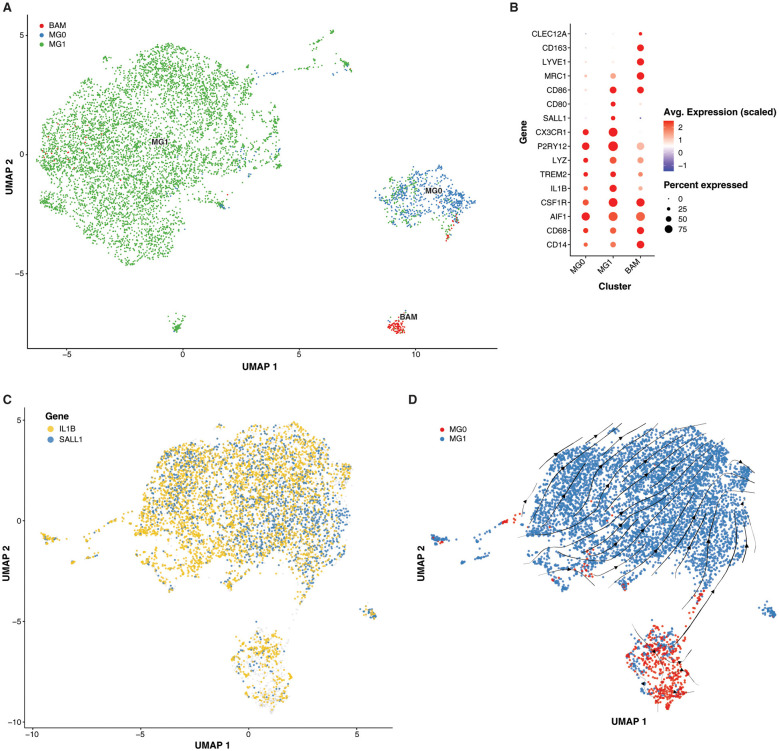
Transcriptomic profile of microglial lineage cells. **(A)** UMAP visualization of microglial clusters (MG0, MG1, and BAM) identified by single-cell RNA-seq. Each point represents an individual cell, and colors denote distinct microglial subpopulations, illustrating transcriptional heterogeneity among microglial cells. **(B)** Dot plot summarizing expression of representative microglia-enriched marker genes across clusters. Dot size indicates the percentage of cells expressing each gene, and color intensity represents scaled average expression levels. The BAM populations were not included in panels C and D, as BAM may have a monocyte origin. **(C)** UMAP feature plot showing expression of *IL1B* (yellow) and *SALL1* (blue) in the MG0 and MG1 clusters, highlighting differential expression of an inflammatory marker and a homeostatic microglia marker across microglial subpopulations. **(D)** RNA velocity trajectories overlaid on the UMAP embedding of MG0 and MG1 clusters, with arrows indicating the predicted direction of microglial state transitions.

### Undifferentiated, stromal, vascular, and epithelial cell populations

An undifferentiated progenitor and non-neuronal cell populations were identified, which would be expected in the developing fetal brain (Figure 8). A UP/I population was characterized by high ribosomal gene expression in early, less committed progenitors. A small population of fibroblasts (FIB) expressed *COL1A2, VIM, DCN*, and *FMOD*. Pericytes (PC) ensheathe capillaries and help to stabilize capillary networks. We identified a single pericyte population that expressed *PDGFRB, MYOF, ABCC9*, and *TAGLN*. Mesenchymal progenitor cells (MPC) were identified by their expression of *VIM, COL1A2, FN1, HMGA2*, and *GREM1*. The endothelial cell (EC) population expressed endothelial cell canonical markers: *PECAM1, VWF, CD34*, and *FLT1*. The choroid plexus (ChP) was identified by its expression of *TTR* and *CLDN 3/4*. Finally, ependymal cells (Ep) were characterized by their expression of *FOXJ1, RFX2/3, DNALI1*, and *CETN2*.

**Figure 8:**
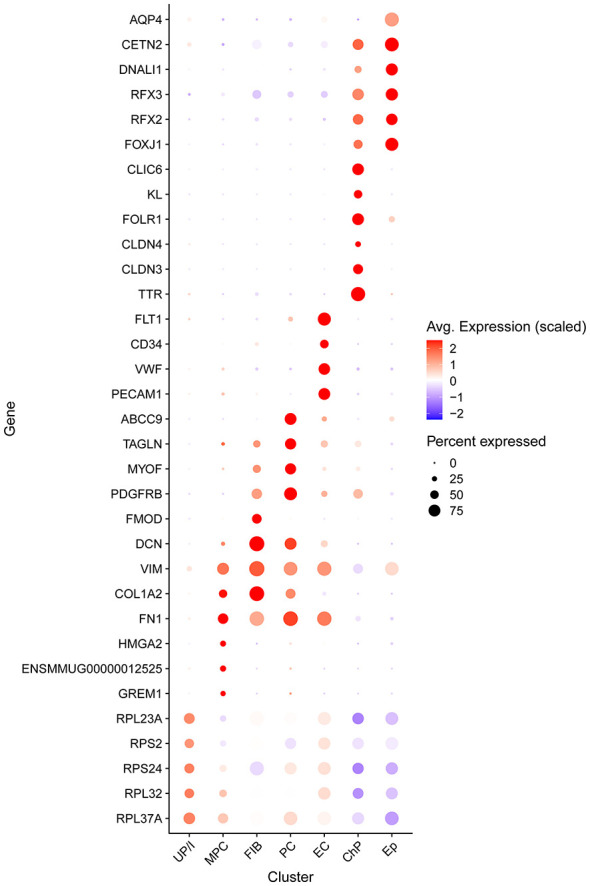
Transcriptomic profile of non-neuronal minor lineages. Dot plot summarizing the expression of canonical marker genes across the undifferentiated progenitor/intermediate (UP/I), mesenchymal progenitor cells (MPC), fibroblasts (FIB), pericytes (PC), endothelial cells (EC), choroid plexus (ChP), and ependymal cells (Ep). Dot size indicates the percentage of cells expressing each gene, and color intensity represents scaled average expression levels.

### Cross-species analysis

We performed a cross-species analysis to compare the single cell populations in our atlas to ones defined in a human fetal single nucleus RNA-Seq atlas of the mid-brain (Supplementary Figure S9). We chose the Brain Cell Atlas as our comparator because it is a comprehensive reference atlas of brain cells throughout major developmental stages (Chen et al., 2024). From this large atlas, we narrowed our dataset for comparison to one labeled “midbrain.” In this comparison, we observed that cell types from our atlas matched major lineages defined in this human atlas. Specifically, there was high concordance between pigtail macaque cell types and corresponding astrocytes [AST0 (72%), AST1 (87%), AST2 (83%)], vascular cells [EC (63%), FIB (91%), and PC (86%)], oligodendrocyte precursors [OPC (99%), COP1 (93%), and OL1 (76%)], microglia [MG0 (63%), MG1 (99%), and BAM (95%)], and “splatter”/neuronal [CGE (70%), MGE (91%), MGE-IN (97%), ExN (98%), and IPC (92%)] lineages.

## Expression of viral entry receptors

As many viruses are known to infect the fetal brain, we investigated the RNA expression of well-established or putative viral entry receptors, co-factors, or enzymes associated with congenital and neonatal neuroinvasive infectious diseases (Figure 9: JEV, TBEV, WNV, ZIKV, VZV, HSV-1/2; Figure 10: CMV, CHIKV, CV, HIV, NiV, SARS-CoV). We focused on genes that were highly upregulated in specific fetal brain cell types, reasoning that these expression patterns might reveal cellular vulnerabilities to viral infection. Notably, BAM and MG1 in the late-gestation fetal brain expressed at least one viral entry receptor exploited by multiple neurotropic viruses, including West Nile virus (WNV; *MRC1, MERTK, TIMD4*, and *ITGAV*), varicella-zoster virus (VZV; *M6PR*), herpes simplex virus-1 and 2 (HSV-1 and 2; *HS3ST1*), coxsackievirus (CV; *CD55*), human immunodeficiency virus (HIV; *CD4*), Nipah virus (NiV; *EFNB2*), and Severe Acute Respiratory Syndrome-related Coronavirus (SARS-CoV-1/SARS-CoV-2; *CTSB, NRP1*). Pericytes within the vascular lineage frequently expressed viral entry receptors, cofactors, or enzymes, including *AXL* (WNV, Zika virus, or chikungunya virus), *IGF2R* (VZV), and *ITGB1* (cytomegalovirus). Finally, endothelial cells expressed viral entry receptors, enzyme or cofactor machinery for Japanese encephalitis virus (*TFRC*) and HSV-1 or−2 *(NECTIN2*).

**Figure 9:**
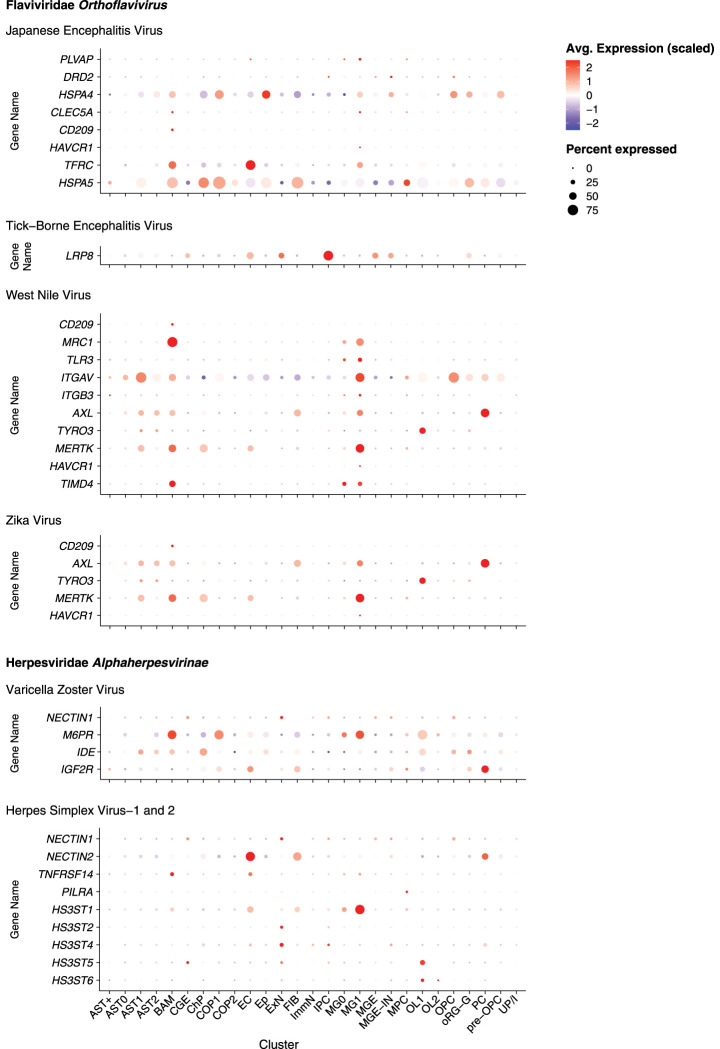
Cell type-specific expression of viral entry receptors, co-factors and enzymes for select pathogenic viruses. Dot plot of genes associated with neuroinvasive congenital or neonatal disease: JEV, TBEV, WNV, ZIKV, VZV, and HSV-1/2.

**Figure 10:**
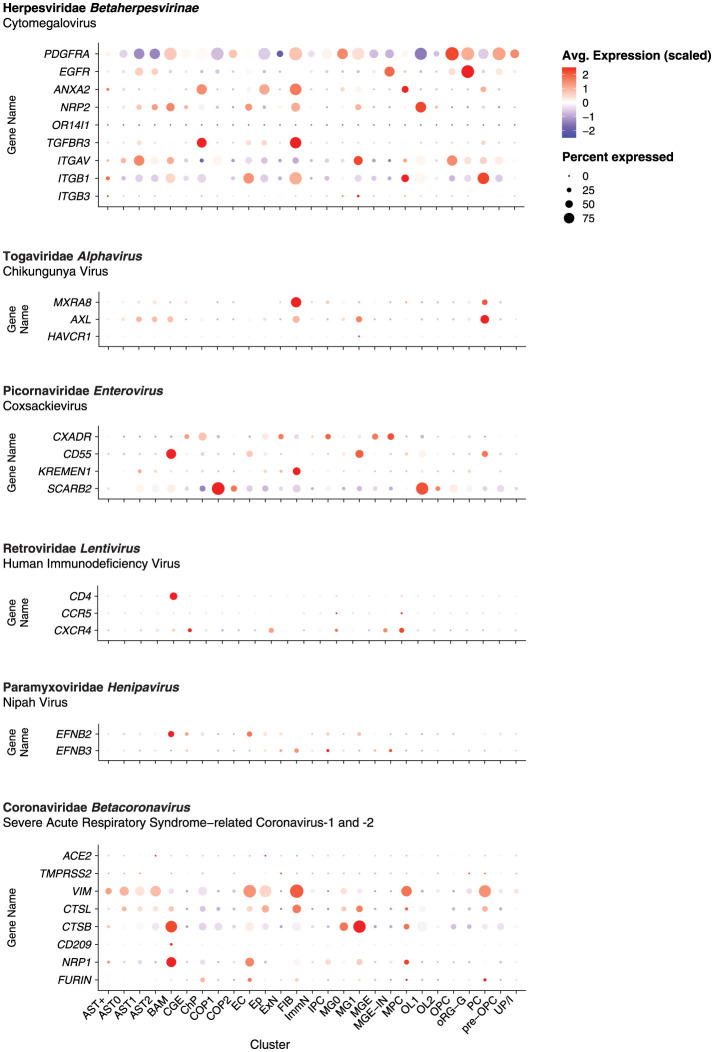
Cell type-specific expression of viral entry receptors, co-factors and enzymes for additional pathogenic viruses. Dot plot of genes associated with neuroinvasive congenital or neonatal disease: CMV, CHIKV, CV, HIV, NiV, and SARS-CoV.

## Discussion

This scRNA-Seq atlas of the periventricular proliferative zone in the late-gestation NHP brain serves as a critical reference for defining normative progenitor, neuronal, and glial populations, in a region of the fetal brain that is vulnerable to injury from preterm birth, hypoxia-ischemia, mechanical ventilation, inflammatory stimuli, and congenital infections (Adams Waldorf et al., 2016; Back, 2017; Abiramalatha et al., 2021; Wharton et al., 2004; Abdu et al., 2025; Anblagan et al., 2016; Adams Waldorf et al., 2018; Tang et al., 2016; Cheeran et al., 2009; Bergström et al., 2021; Codaccioni et al., 2020). The atlas revealed a high degree of transcriptional similarity in the fetal pigtail macaque single cell populations of the periventricular proliferative region with the astrocyte, oligodendrocyte precursors, vascular, microglial, and neuronal lineages of humans. The late timing in gestation and location of sampling makes this atlas rich in maturing neuronal and glial cell populations. A scRNA-Seq reference atlas to describe normal transcriptional programs in the periventricular region may provide insight into how hypoxic, inflammatory, and other insults disrupt neuroprogenitor development and lead to ventriculomegaly, periventricular leukomalacia, and/or long-term neurodevelopmental deficits (Woodward et al., 2012; Pappas et al., 2018).

Similarities in the single-cell populations between the macaque and human brain in late gestation are expected; both human and nonhuman primate brains are characterized by early symmetric divisions of neural progenitor that expand the progenitor pool, followed by asymmetric divisions that generate neurons and glia organized into cortical columns (Sidman and Rakic, 1973; Pontious et al., 2008; Rakic, 1995; Martinez-Cerdeno et al., 2006; Kriegstein et al., 2006; Bystron et al., 2008). Radial glia scaffold the migration of cortical neurons during a prolonged prenatal period of neurogenesis and neuronal maturation, resulting in the complex, gyrencephalic brain characteristic of primates (Kriegstein et al., 2006), which contrasts with the lissencephalic brains of other species. In the late-gestation fetal macaque brain, we identified key subpopulations of radial glia and neural progenitors: actively cycling pre-OPC (*MKI67*+) and oRG-G; *HOPX*+ astrocytes derived from oRG-G; and IPC (*TBR2*+) with a trajectory toward ExN differentiation. At this time in gestation, neurons remained relatively immature with poor expression of markers characteristic of mature interneurons (MGE: *PVALB, SST*; CGE: *VIP, RELN*). Consistent with prior developmental studies, single cell populations within the astrocyte and oligodendrocyte lineages displayed partially redundant transcriptional programs, with subtle distinctions driven by cellular origin, niche, or timing of differentiation (Schroeder et al., 2025; Zhou et al., 2025; Gao et al., 2023; Foerster et al., 2024; Kessaris et al., 2006; Marques et al., 2018, 2016; Dennis et al., 2024; Perlman et al., 2020; Chamling et al., 2021; van et al., 2022). In addition, a quiescent UP/I population was positioned upstream of multiple, actively cycling populations; the high ribosomal gene expression in this population is similar to other descriptions of early, less committed progenitor populations (Chau et al., 2018; Ni et al., 2025).

There are several advantages to this late gestation scRNA-Seq atlas of the periventricular proliferative region over other human and NHP atlases. Although human fetal brain atlases provide broader anatomical coverage, they have been mainly restricted to early gestation (Qian et al., 2025; Zeng et al., 2023; Braun et al., 2023; Li et al., 2018), used a spatial (Qian et al., 2025) or single-nucleus RNA-Seq (snRNA-Seq) approach (Velmeshev et al., 2023), or focused on other areas of the brain (Toh et al., 2025). When third-trimester human fetal brains have been studied, the tissue is collected post-mortem and analyzed using spatial or snRNA-Seq. Two rhesus macaque fetal brain atlases have been described, but these were collected from the first half of gestation and focused on progenitor and neuron lineages (Micali et al., 2023; Xu et al., 2024). Other atlases have used combinations of fetal brain explants and human induced pluripotent stem cell-derived mixed neural cultures to define pathways induced during *ex vivo* stimulation with Zika virus (Stokes et al., 2025). Mouse scRNA-Seq atlases of the fetal brain also exist (Brooks et al., 2025), but murine neurodevelopment occurs over a shorter timeline than in humans and nonhuman primates. Therefore, the nonhuman primate is a powerful model of human brain development because it shares fundamental aspects of cortical lamination, progenitor biology, and an extended gestational maturation that closely mirror human neurodevelopment.

This single-cell RNA sequencing atlas provides an important resource for identifying fetal brain cell types that may be vulnerable to neuroinvasive viruses associated with congenital and neonatal infections. For viruses that exploit multiple entry pathways, such as human cytomegalovirus, our data suggests that numerous cell types in the late-gestation fetal brain may be permissive to infection. Notably, BAM, microglia, and cells in the vascular lineage appear particularly susceptible to a broad range of viruses; their strategic localization at fetal brain barrier regions suggests they may serve as an early cellular interface facilitating productive infection and neuroinvasion. In our late-gestation atlas, a key viral entry receptor for ZIKV (*AXL*) was expressed in pericytes, microglia, fibroblasts, and astrocytes, but not pre-OPC, a key target population in the fetal brain; dynamic changes in pre-OPC expression over gestation may explain reduced AXL expression in pre-OPCs, and potentially, a lower susceptibility to ZIKV infection in the third trimester (Nowakowski et al., 2017, 2016). More broadly, this atlas enables systematic screening for viral entry receptors in the fetal brain associated with emerging viruses that could cause congenital infection. Finally, a notable strength of this atlas is the inclusion of BAM populations, which are often underrepresented in human fetal brain datasets but play critical roles in shaping neurodevelopmental responses to inflammatory and infectious exposures.

The use of the pigtail macaque, whose gestational timeline and placentation parallel human development (Li et al., 2021, 2025; Adams Waldorf et al., 2011), further enhances the translational relevance of this atlas for modeling neurodevelopmental disease and congenital infection. While histopathologic and imaging approaches have described the gross architecture of periventricular injury, they cannot resolve the cellular subpopulations affected or the mechanisms by which transcriptional programs are perturbed. This scRNA-Seq atlas provides a resource to overcome this limitation by identifying normal cellular transcriptional states in late gestation and by uncovering transcriptional networks that shape local immune and microglial activation. A limitation of this study is the collection of tissue from only the late second and third trimesters, which provides a temporal snapshot from late in gestation but leaves earlier neurogenic waves unexplored. This scRNA-Seq atlas is also focused on the periventricular proliferative zone and does not profile other important brain regions. There are also several species differences between macaque and human neurodevelopment to consider: slower postsynaptic density protein accumulation in the human fetal brain and distinct postnatal trajectories for specific lineages and regions after birth (Zhu et al., 2018; Wang et al., 2023). Finally, while scRNA-Seq defines cellular diversity at high resolution, functional validation through *in situ* hybridization, spatial transcriptomics, and lineage tracing is important to confirm inferred trajectories.

In summary, this late-gestation scRNA-Seq atlas of the periventricular proliferative region provides a transcriptional map of a highly vulnerable area of the developing primate brain. The late second and third trimesters represent a critical window of human brain development, during which the periventricular proliferative zone and adjacent intermediate and white matter regions undergo rapid expansion, gliogenesis, and maturation of cortical circuitry. Maternal infectious exposures and immune activation have been linked to fetal brain injury using murine and NHP models, as well as through epidemiologic studies of children (Han et al., 2021; Estes and McAllister, 2016; Ryan and Bauman, 2022; Bauman and Van de Water, 2020; Al-Haddad et al., 2019a,b). Fetal exposure to a viral mimetic [poly(I:C), polyriboinosinic–polyribocytidylic acid) during pregnancy has been associated with disruptions in neuroprogenitor proliferation and migration, altered cortical layering and glial organization, brain growth, and deficits in attention and behavior in offspring (Gillespie et al., 2024; Carpentier et al., 2013; O'Loughlin et al., 2017; Soumiya et al., 2011; Canales et al., 2021; Hubbard et al., 2025; Vlasova et al., 2021; Bauman et al., 2014). This atlas enables comparison of normal and perturbed developmental trajectories in NHP models, and these insights may point to pathways that could be leveraged for neuroprotection or repair. An interesting finding in our atlas is that glial maturation is supported by redundant transcriptional programs, in which both canonical and alternative transcriptomic pathways independently reach mature oligodendrocyte and astrocyte states. Future studies using this atlas as a benchmark can determine how prenatal insults of varying types (e.g., inflammation, infection, preterm birth) perturb single-cell populations in this critical area of the fetal brain.

## Methods

### Ethics statement

All nonhuman primate experiments were performed in strict accordance with the recommendations in the Guide for the Care and Use of Laboratory Animals of the National Research Council and the Weatherall report, “The use of non-human primates in research.” The Institutional Animal Care and Use Committee (IACUC) of the University of Washington (UW) approved the study's protocols (#4165-01, last approval date: 02/20/2026; #4165-02, last approval date: 05/07/2026). All surgeries were performed under general anesthesia, and all efforts were made to minimize pain and distress.

### Experimental design

Nine healthy, pregnant pigtail macaques were enrolled as uninfected controls for two different experimental protocols. One group underwent surgical catheterization of choriodecidual and amniotic catheters; three of four animals received choriodecidual and intra-amniotic saline infusions through these catheters, and the fourth animal received no inoculation due to twisting of the catheters (*N* = 4). This was a control group for a Group B Streptococcus study. A second group received five subcutaneous inoculations of media along the forearm to serve as controls for a study of Zika virus pathogenesis (*N* = 5). Gestational ages at tissue collection spanned the late second and third trimester (128.4–158.4 days of gestation; term gestation = 172 days). After delivery by Cesarean section, tissues from the ventricular zone, subventricular zone, periventricular white matter, and deep white matter were collected from the fetal brain. Samples were collected in Hibernate-E medium (Thermo-Fisher, Waltham, MA, A1247601) on wet ice, and single-cell dissociation was performed within 1 h after collection.

### Single cell RNA-Seq and bioinformatics pipeline

#### Single cell dissociation

The right hemisphere of each brain was sectioned coronally at approximately 0.5 cm intervals. From the coronal sections, brain samples for scRNA-Seq were manually removed to prioritize sampling of the lateral ventricular wall (ventricular zone, VZ), subventricular zone (SVZ), and adjacent white matter. This protocol evolved over the course of the study. Initially, only the periventricular region of the parietal cortex was selectively sampled, but we obtained insufficient cells to complete the scRNA-Seq protocol. Therefore, we shifted to sampling full-thickness portions of the parietal cortex, including the lateral ventricle wall, VZ, SVZ, deep white matter, and adjacent white/cortical gray matter. Later, we carved out the lateral ventricle wall, VZ, SVZ, and deep white matter from the frontal, parietal, temporal, and occipital coronal sections, excluding cortical gray matter and deep nuclei. Most samples were obtained using the latter approach.

#### Isolation of cells from the fetal brain

Fetal brain tissues were placed into cold HBSS with calcium and magnesium (Thermo-Fisher, 14025092) and finely chopped into approximately 0.2 mm3 cubes using sterile scissors. Tissue fragments were enzymatically digested in 2 mL of a papain digestion buffer. The digestion buffer consisted of 2.5 U/mL papain (Worthington, Lakewood, NJ, #LS003126), 250 U/mL DNase I (Qiagen, Hilden, Germany, #79254), and 10 mM HEPES (Thermo Fisher, Waltham, MA, #15630130), prepared in HBSS without calcium or magnesium (Thermo Fisher, Waltham, MA, #14175095). Digestion was carried out at 37°C for 20 min, and the tube was gently inverted every 5 min to mix.

Following digestion, the digestion buffer was quenched with 6 mL of cold quenching buffer. L-15 media consisting of 440 mL Leibovitz L-15 media (Thermo-Fisher, Waltham, MA, #21083027), 53 mL purified H2O, 5 mL of 1M HEPES (Thermo Fisher, #15630130), and 0.4% BSA (Fisher Scientific, Waltham, MA, #50-121-5315) was mixed with 250 U/mL DNase I (Qiagen, #79254) and 0.5 mg/mL trypsin inhibitor (Sigma-Aldrich, Burlington, MA, T2011) to make the quenching buffer. Following quenching and centrifugation at 200 × g for 5 min at room temperature, the supernatant was removed, and an additional 1 mL of quenching buffer was added. Using a P-1000 micropipette, the sample was gently pipetted up and down to disperse the tissue into a single cell suspension after approximately 15 cycles.

To wash the sample, the cells were transferred to a new 50 mL tube and resuspended in 40 mL of resuspension buffer, prepared with the same materials as the quenching buffer, but without trypsin inhibitor. The suspension was then filtered with a 40-μm cell strainer (Fisher Scientific, #22-363-547) and centrifuged at 200 × g for 10 min at room temperature. The supernatant was removed, and the cells were resuspended to 10 mL of resuspension buffer. Cells were then counted, and cell viability was assessed using trypan blue (Fisher Scientific, #MT25900CI). Single-cell libraries were prepared immediately following dissociation.

#### Sequencing

10x Chromium single-cell libraries were prepared according to the standard protocol outlined in the manual (Chromium Next GEM Single Cell 3′ kits v3.1: dual index, 10X Genomics, Pleasanton, CA). Briefly, we loaded single-cell suspensions, 10x barcoded gel beads, and reagents onto a “Chromium Single Cell A Chip” to generate Gel Bead-In-EMulsions (GEMs) using the 10X Chromium Controller (10X Genomics, Pleasanton, CA). Reverse transcription of mRNA occurred inside each GEM, followed by amplification of cDNA and preparation of sequencing libraries according to manufacturer instructions. Quality control was performed in multiple steps to check the quality of the cDNA and the library using the Agilent 2200 TapeStation system (Agilent Technologies, Santa Clara, CA). Finally, shallow sequencing was performed using a NextSeq 2000 (Illumina Inc., San Diego, CA) to evaluate the percentage of reads mapping to the reference genome, the distribution of GC content, and sequencing depth (number of reads per cell). Based on this information, we determined if the cDNA library was of sufficient quality to send for deep sequencing and whether an adjustment to the amount of any samples was necessary to achieve a greater number of reads per cell. Deep sequencing of the cDNA library was performed on a NovaSeq (Illumina Inc., San Diego, CA) to achieve approximately 40,000 reads per cell.

#### Genome alignment

Raw FASTQ files were processed using Cell Ranger v7.2.0 software (10x Genomics, Pleasanton, CA, USA). Reads were aligned to the rhesus macaque genome (Mmul_10, GenBank Assembly ID: GCA_003339765.3), which has a contiguous assembly and is more completely annotated than the pigtail macaque genome. Because the rhesus and pigtail macaque are closely related species with high conservation of coding and regulatory regions, alignment to the rhesus macaque reference genome enabled robust transcript detection while minimizing gene annotation loss that would occur using the less complete pigtail macaque assembly.

#### Quality control, normalization, and harmonization

For each of the nine samples, both the raw and CellRanger-filtered feature-barcode matrices were loaded into R (v4.5.2) using Seurat (v5.5.0) [Seurat v5 (Satija Lab, New York Genome Center], (Hao et al., 2024, 2021; Stuart et al., 2019; Butler et al., 2018; Satija et al., 2015) and DropletUtils (v1.30.0) (Lun et al., 2019). Empty droplet removal was performed on the raw barcode matrix using emptyDrops (DropletUtils; FDR < 0.01, seed = 42); if fewer than 100 cells were called, the sample fell back to the CellRanger-filtered matrix. Ambient RNA contamination was estimated and corrected per-sample using SoupX (v1.6.2) (Young and Behjati, 2020), with preliminary cluster assignments derived from a standard Seurat workflow (NormalizeData, FindVariableFeatures, ScaleData, RunPCA, FindNeighbors, FindClusters at resolution 0.5); samples with fewer than 100 cells were excluded from SoupX correction. Per-sample Seurat objects were then subjected to an initial quality filter retaining cells with more than 200 detected genes and mitochondrial read fraction below 5%. All samples were merged into a single Seurat object, and doublets were identified per sample using scDblFinder (v1.24.10) (run on the merged SingleCellExperiment object with samples = “orig.ident,” seed = 42). A final quality filter was applied to the merged object, retaining singlets with 200–7,500 detected genes and mitochondrial fraction below 2.5%, yielding 35,836 high-quality cells across nine samples (87.6% retention from emptyDrops-called cells). Counts were normalized using SCTransform (Choudhary and Satija, 2022) with mitochondrial percentage regressed out, followed by PCA (50 PCs) and cross-sample batch correction with Harmony (v1.2.4) (dims 1:30, theta = 2, max iterations = 20). UMAP embeddings were computed on the Harmony-corrected PCA space (30 neighbors, min.dist=0.3), and graph-based clustering was performed with FindNeighbors/FindClusters (resolution 0.5) (Korsunsky et al., 2019). After cluster annotation, scCustomize was then used to generate final UMAPs (Marsh, 2021).

#### Trajectory inference

To quantify RNA velocity, spliced, unspliced, and ambiguous read counts were obtained for each of the nine samples by running velocyto (v0.17.17) run10x on the Cell Ranger-generated BAM file using the corresponding genome annotation GTF, producing per-sample loom files. Loom files were loaded and concatenated using scanpy (v1.11.2) and scVelo (v0.3.3); barcodes were cleaned from velocyto's sample:barcode format and matched to the QC-filtered Seurat object to transfer cell type annotations and Harmony-corrected UMAP coordinates. Velocity was computed in two configurations: (1) the full 35,836-cell atlas and (2) five biologically stratified lineage subsets. For each subset, counts were filtered and normalized with scv.pp.filter_and_normalize (min_shared_counts = 20, n_top_genes = 2,000) and first- and second-order moments were computed with scv.pp.moments (n_pcs = 30, n_neighbors = 30). RNA velocity was estimated in stochastic mode (scv.tl.velocity, scv.tl.velocity_graph), followed by calculation of velocity pseudotime, velocity confidence, and per-cell-type top velocity genes (minimum Spearman correlation 0.3). Fate analysis was performed with CellRank 2 (v2.2.0): for each lineage subset, a combined transition kernel was constructed as a weighted sum of 80% VelocityKernel and 20% ConnectivityKernel. Macrostates were identified using the GPCCA estimator via Schur decomposition (n_components = 20) with the number of states selected by the largest eigengap in the real Schur spectrum; terminal and initial states were designated automatically via predict_terminal_states/predict_initial_states, or manually fixed for the microglia subset (MG0, MG1). Cell-level fate probabilities were computed by solving the absorbing Markov chain system (I – T_QQ)B = T_QR using direct LU decomposition (scipy.sparse.linalg.spsolve), with macrostate memberships used as a soft approximation when cell separation was insufficient. Lineage driver genes were identified via g.compute_lineage_drivers() (top 50 per fate by Spearman correlation with fate probability). Cluster-specific drivers were independently computed for every cell type by Spearman correlation of normalized expression against binary cluster membership across all variable genes, with Benjamini–Hochberg false discovery rate correction; genes with *q* < 0.05 were considered significant. Gene expression trends along velocity pseudotime were visualized using a custom weighted binning approach (30 bins, cells weighted by fate probability > 0.05) applied independently to each lineage, with a smoothed trend line overlaid on individual cell scatter.

#### Viral receptor analysis

We conducted a literature review to identify established and putative viral entry receptors, cofactors, and enzymes for 14 neurotropic and systemic viruses with known or suspected capacity to infect the fetal or neonatal brain: Japanese encephalitis virus (JEV), tick-borne encephalitis virus (TBEV), West Nile virus (WNV), Zika virus (ZIKV), varicella-zoster virus (VZV), herpes simplex virus types 1 and 2 (HSV-1 and HSV-2), cytomegalovirus (CMV), chikungunya virus (CHIKV), coxsackie virus (CV), human immunodeficiency virus (HIV), Nipah virus (NiV), severe acute respiratory syndrome coronavirus (SARS-CoV-1), and SARS-CoV-2. We searched PubMed and Google Scholar databases using targeted queries combining each virus name with terms such as “entry receptor,” “cofactor,” “cell tropism,” and “fetal infection.” We compiled all identified entry-associated molecules into a curated reference list, prioritizing peer-reviewed literature and integrating both well-established receptors (e.g., ACE2 for SARS-CoV-2) and emerging putative factors identified in recent studies. Using this comprehensive molecular reference set, we constructed dot plots to visualize the expression profiles of these identified genes and their encoded proteins across distinct cell types in the fetal brain.

#### Cross-species integration and comparison

We performed a cross-species atlas comparison to human data contained within the Brain Cell Atlas, because it is a comprehensive reference atlas of brain cells, which includes single-cell data from 70 human and 103 mouse studies of the brain throughout major developmental stages (Chen et al., 2024). From this large atlas, we narrowed our dataset for comparison to one labeled “midbrain,” which contains scRNA-Seq data from the ventral midbrain of human fetal and mouse studies. Next, we removed data from mouse studies to restrict the cross-species comparison to human vs. pigtail macaque. We performed Seurat label transfer onto the human reference and Harmony integration with species and study as covariates to do the actual cross species analysis. Concordance between cell types and lineages was evaluated by determining the percentage of overlap of predicted cell type labels after Seurat label transfer.

## Data Availability

The datasets presented in this study can be found in online repositories. The names of the repository/repositories and accession number(s) can be found below: https://www.ncbi.nlm.nih.gov/geo/ (GSE305531). The data can be explored using the UCSC Cell Browser: https://cells-test.gi.ucsc.edu/?ds=nemestrina-fetal-svz.
