# Supplementary material for: A single-cell transcriptomic atlas of the periventricular proliferative zone in the late gestation fetal brain in the pigtail macaque
Source: Front Neurosci. 2026 Aug 3;20:1785001. doi: 10.3389/fnins.2026.1785001 (PMC13478084; doi:10.3389/fnins.2026.1785001)
Supplement: Supplementary file 1 [file Data_Sheet_1.pdf]

SUPPLEMENTAL INFORMATION

Table of Contents

TABLE S1. CELL TYPE COUNTS 2

TABLE S2. QUALITY CONTROL STATISTICS FOR EACH SAMPLE 3

FIGURE S1. PROPORTION OF SINGLE-CELL POPULATIONS BY TYPE OF TREATMENT 4

FIGURE S2. PROPORTION OF SINGLE-CELL POPULATIONS BY FETAL SEX 5

FIGURE S3. CELL CYCLE SCORING BY CELL TYPE 6

FIGURE S4. RNA VELOCITY TRAJECTORIES OF NEURONAL LINEAGE CELLS 7

FIGURE S5. DISTRIBUTION OF MICROGLIAL SINGLE CELL POPULATIONS BY SAMPLE 8

FIGURE S6. GENE EXPRESSION OF HOMEOSTATIC AND TISSUE-RESIDENT IDENTITY MARKERS IN MICROGLIAL POPULATIONS 9

FIGURE S7. GENE EXPRESSION OF INFLAMMATORY ACTIVATION AND INTERFERON RESPONSE MARKERS IN MICROGLIAL POPULATIONS 10

FIGURE S8. PSEUDOTIME EXPRESSION OF *IL1B* AND *CD86* IN THE MICROGLIA LINEAGE 11

FIGURE S9. CROSS-SPECIES ATLAS COMPARISON BETWEEN HUMAN FETAL MID-BRAIN AND PIGTAIL MACAQUE PERIVENTRICULAR WHITE MATTER 12

Table S1. Cell Type Counts

Cell Type	Count	Percent
MG1	6635	18.51
OPC	5069	14.14
CGE	2433	6.79
AST2	2188	6.11
COP1	1960	5.47
oRG-G	1873	5.23
EC	1850	5.16
OL1	1802	5.03
Ep	1555	4.34
AST1	1472	4.11
pre-OPC	1398	3.9
UP/I	1317	3.68
AST0	754	2.1
ExN	667	1.86
PC	663	1.85
MG0	657	1.83
MPC	631	1.76
OL2	612	1.71
MGE	530	1.48
ImmN	449	1.25
AST+	323	0.9
IPC	278	0.78
MGE-IN	168	0.47
FIB	165	0.46
BAM	141	0.39
COP2	133	0.37
ChP	113	0.32
**Total**	35836	100

This table shows the single cell counts and percent of total cells for each single cell population. Cell populations are ordered from most to least abundant. Abbreviations: AST, astrocyte; AST+, reactive astrocyte; BAM, border-associated macrophage; CGE, caudal ganglionic eminence lineage; ChP, choroid plexus; COP, committed oligodendrocyte progenitor; EC, endothelial cell; Ep, ependymal cell; ExN, excitatory neuron lineage; FIB, fibroblast; ImmN, immature neurons; IPC, intermediate progenitor cell; MG, microglia; MGE, medial ganglionic eminence lineage; MGE-IN, MGE-derived interneuron; MPC, mesenchymal progenitor cell; OL, oligodendrocyte; OPC, oligodendrocyte progenitor cell; oRG-G, outer radial glia undergoing gliogenic differentiation; PC, pericyte; UP/I, unipolar brush cell/interneuron; and vRG, ventricular radial glia.

Table S2. Quality Control Statistics for Each Sample

Sample	Raw Barcodes	Cells Remaining After Empty Drops	Basic QC	Doublets Removed	Final	% Retained
CTRL1	480402	2298	1861	86	1779	77.4
CTRL2	964550	3954	2873	159	2602	65.8
CTRL3	1446279	4997	4676	306	4368	87.4
CTRL4	548578	1695	1677	71	1593	94
CTRL5	469162	1839	1763	74	1713	93.1
CTRL6	1440570	5888	5821	474	5339	90.7
CTRL7	699146	3366	2838	155	2655	78.9
CTRL8	1176258	9834	9742	847	8984	91.4
CTRL9	595703	7035	7029	249	6803	96.7
Total	7820648	40906	38280	2421	35836	87.6

This table shows the quality control metrics of our analysis pipeline on a per-sample basis progressing from raw cell counts through the final number of cells kept. Each column shows cell counts at respective stages of filtering. Doublets Removed indicates how many cells were identified as doublets. “Percent Retained” is a ratio of the “Final Counts” divided by the “Cells Remaining After Empty Drops” columns.

Figure S1. Proportion of Single-Cell Populations by Type of Treatment

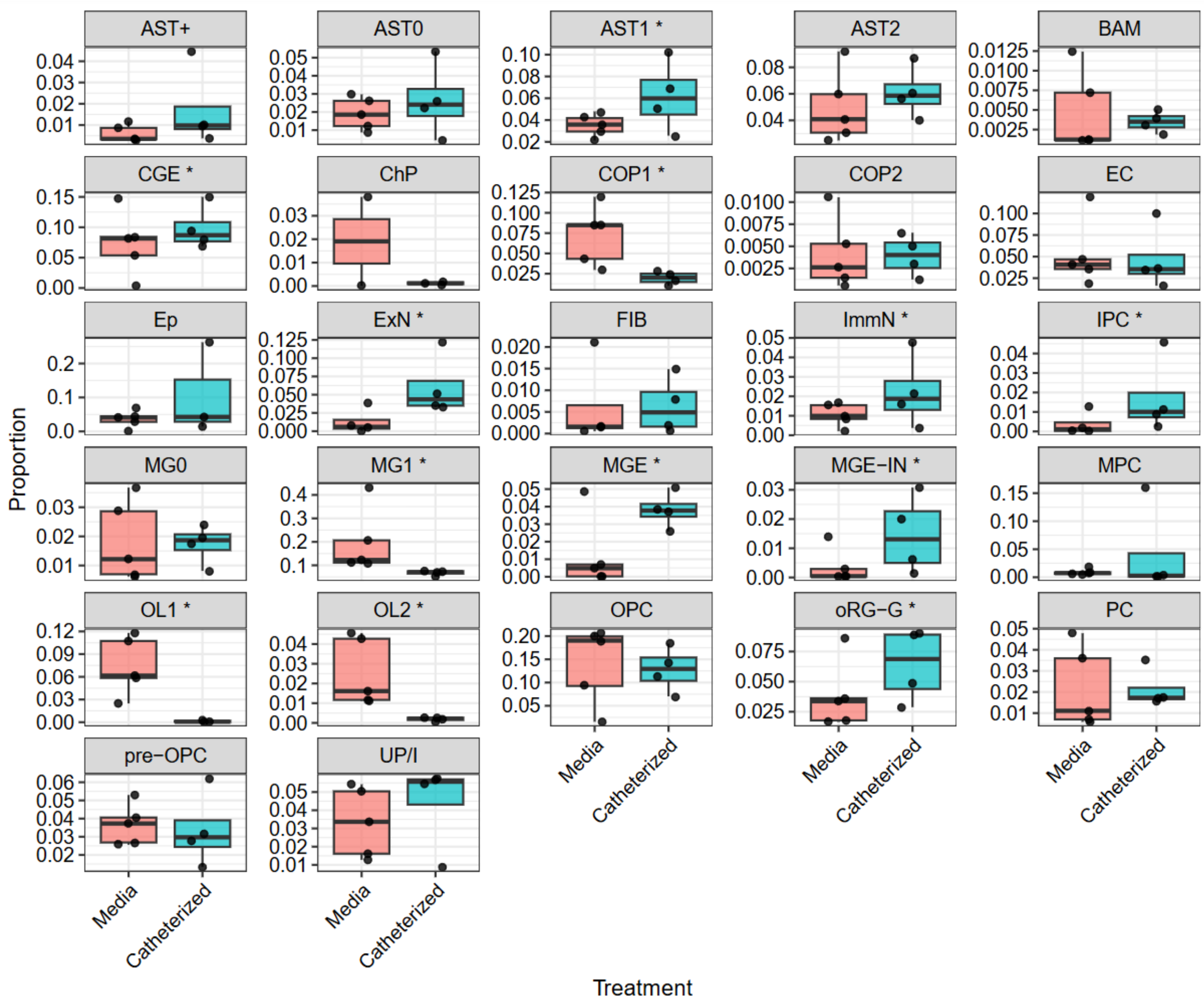

This figure illustrates the proportion of single-cell populations (y-axis) out of all single-cell populations for each type of control: media or catheterized. Each dot within a bar plot represents a single sample. The boxes show the median and the interquartile range. A star next to the cell type label indicates a significant difference in proportions between the media and catheterized groups using scComp ($p<0.05$). Abbreviations for single cell populations are shown in the legend of Table S1.

Figure S2. Proportion of Single-Cell Populations by Fetal Sex

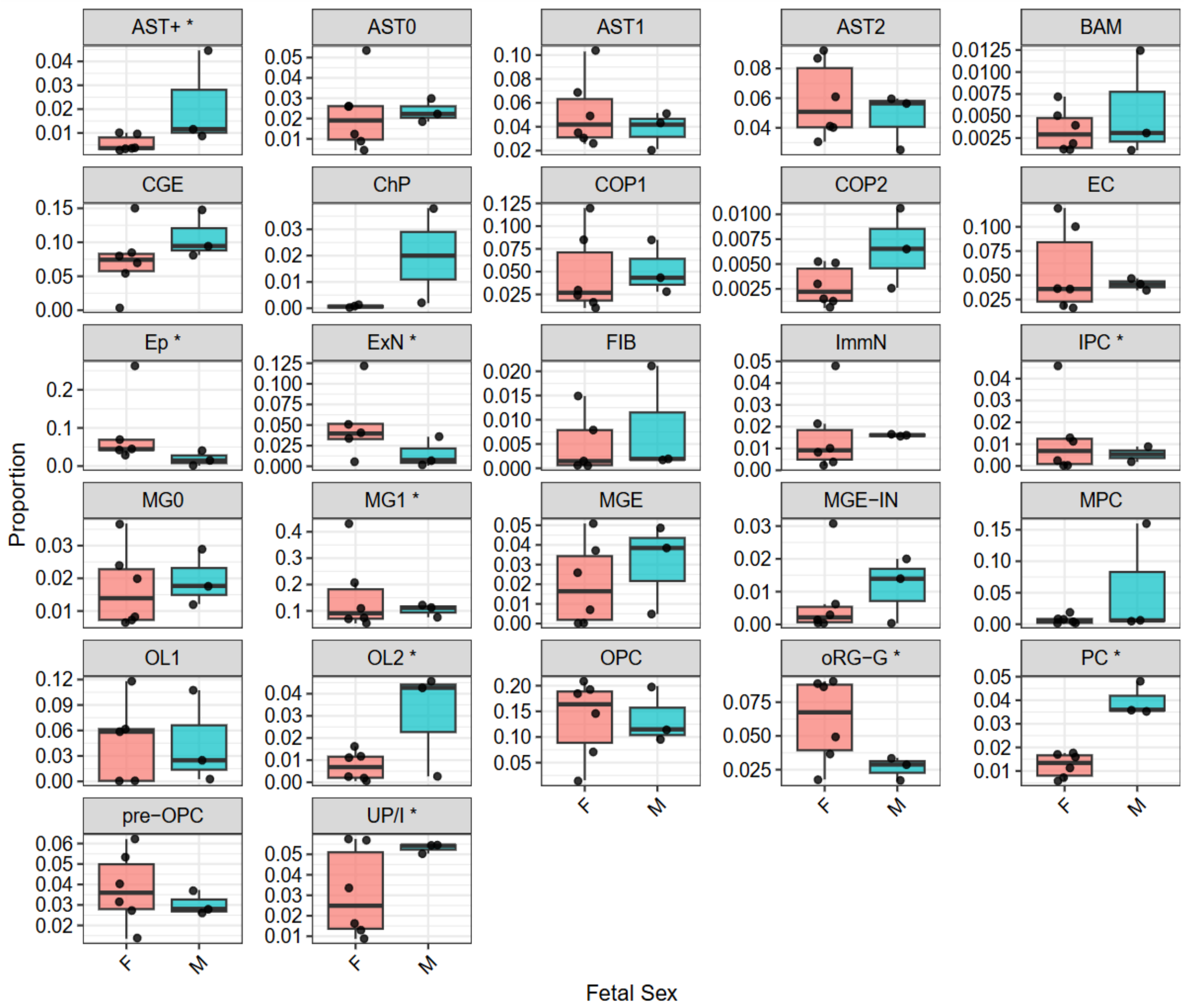

This figure illustrates the proportion of single-cell populations (y-axis) out of all single-cell populations for each fetal sex: female (F) or male (M). Each dot within a bar plot represents a single sample. The boxes show the median and the interquartile range. A star next to the cell type label indicates a significant difference in proportions between the media and catheterized groups using scComp ($p<0.05$). Abbreviations for single cell populations are shown in the legend of Table S1.

Figure S3. Cell Cycle Scoring by Cell Type

A

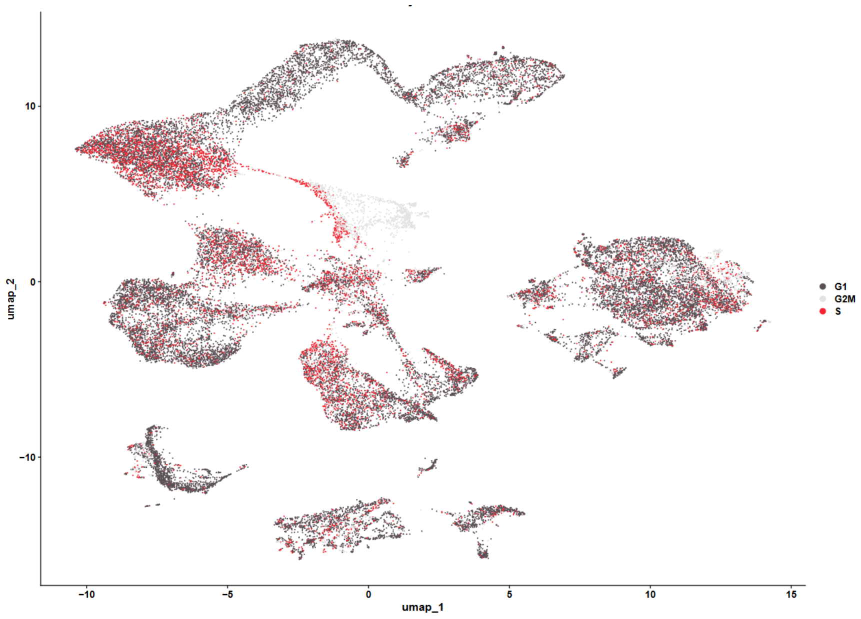

B

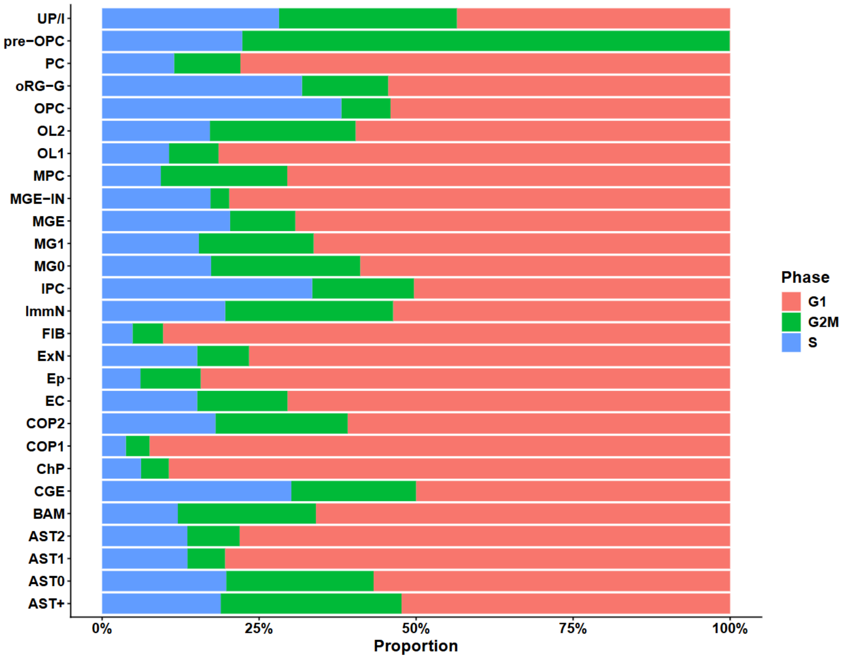

This plot shows the cell cycle scoring of each cell in the UMAP (A) with colored dots indicating S phase (red), G1 (dark gray), and G2M (light gray). In panel B, the proportion of cells in a population that is within each phase is shown for S phase (blue), G1 (red) and G2M (green).

Figure S4. RNA Velocity Trajectories of Neuronal Lineage Cells

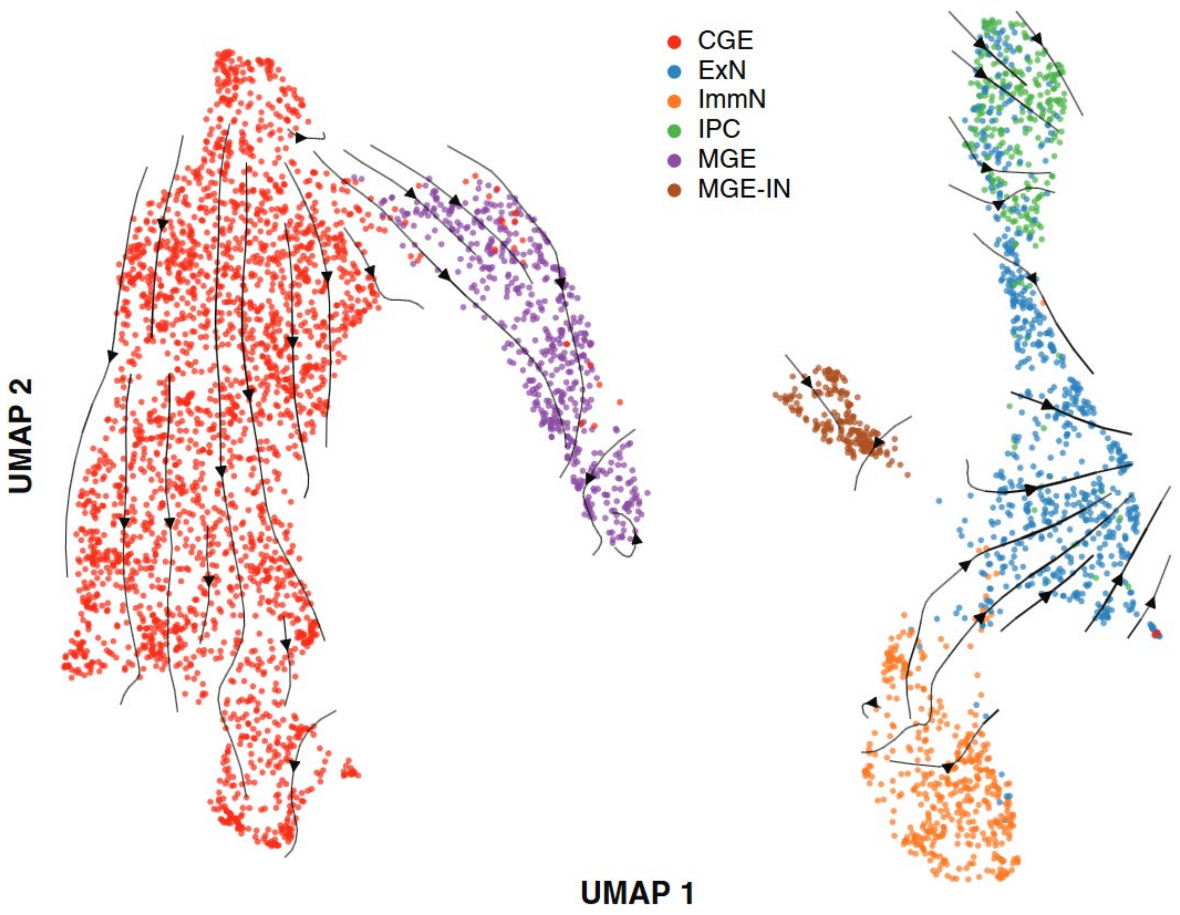

This figure shows the RNA velocity trajectories of neuronal lineage cells in the pigtail macaque fetal brain atlas, with arrows indicating predicted differentiation directions. Cell populations include caudal ganglionic eminence (CGE), excitatory neurons (ExN), intermediate progenitor cells (IPC), immature neurons (ImmN), medial ganglionic eminence progenitors (MGE), and MGE-derived interneurons (MGE-IN).

Figure S5. Distribution of Microglial Single Cell Populations by Sample

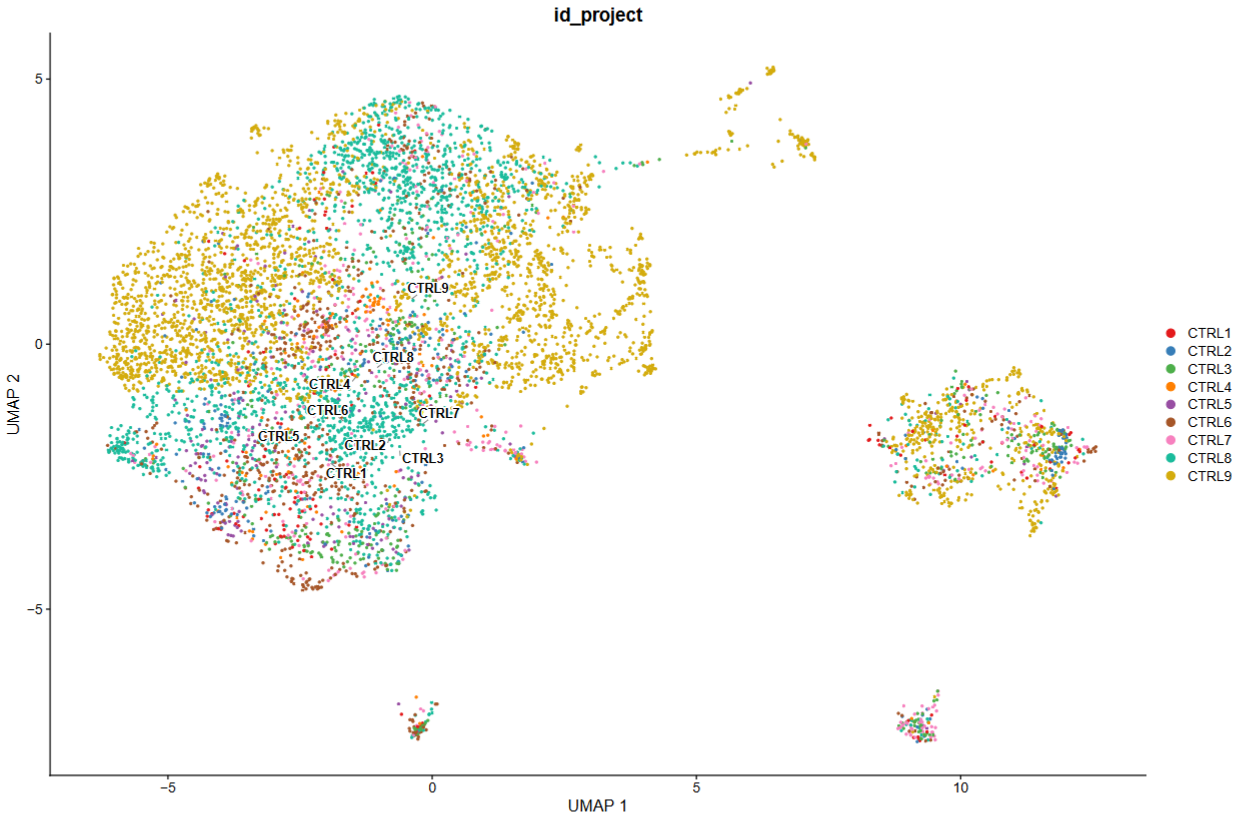

This figure shows the UMAP embeddings of microglial single-cell populations colored by sample identity. Each dot represents a single cell from a single animal demonstrating the distribution and intermixing of samples across clusters.

Figure S6. Gene Expression of Homeostatic and Tissue-Resident Identity Markers in Microglial Populations

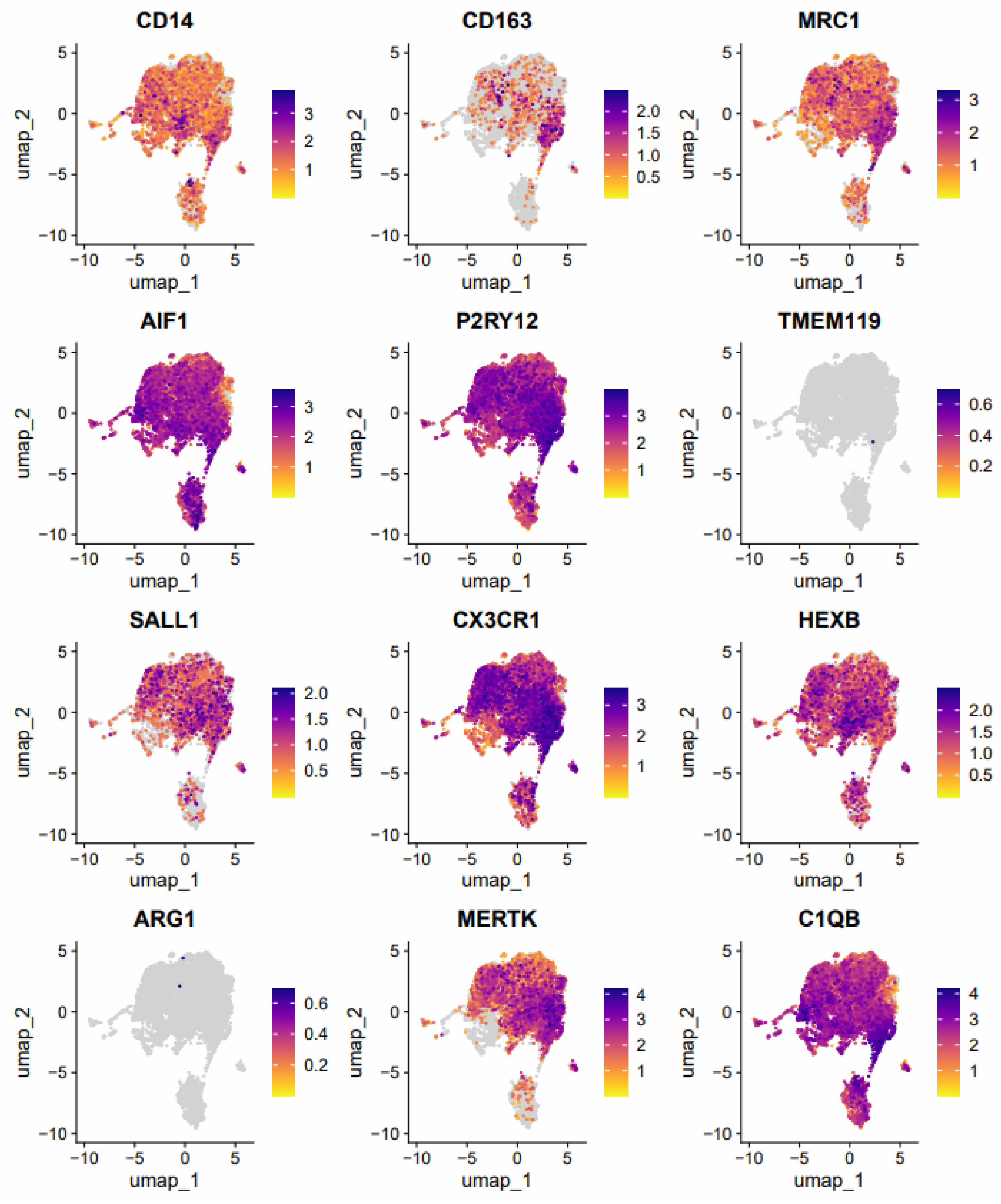

Feature plots show gene expression of homeostatic and tissue-resident identity markers in microglial populations in the fetal pigtail macaque periventricular white matter.

Figure S7. Gene Expression of Inflammatory Activation and Interferon Response Markers in Microglial Populations

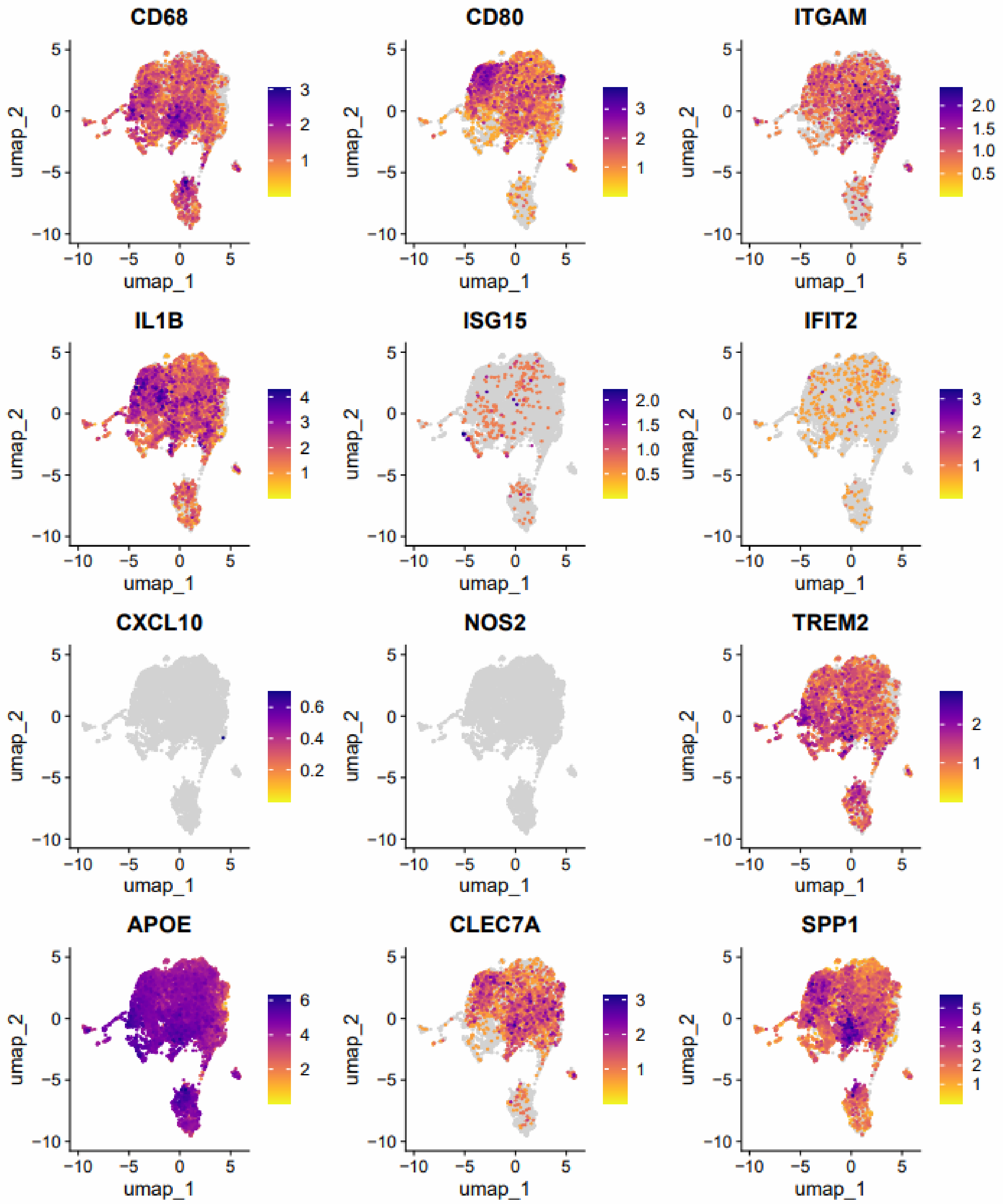

Feature plots show gene expression of inflammatory activation and interferon response markers in the fetal pigtail macaque periventricular white matter.

Figure S8. Pseudotime Expression of *IL1B* and *CD86* in the Microglia Lineage

A

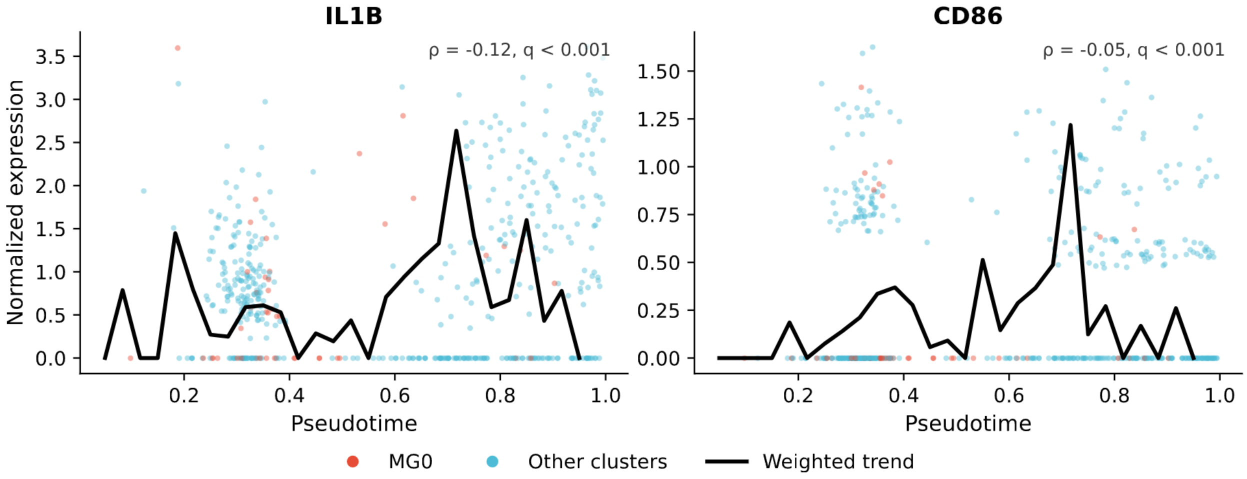

B

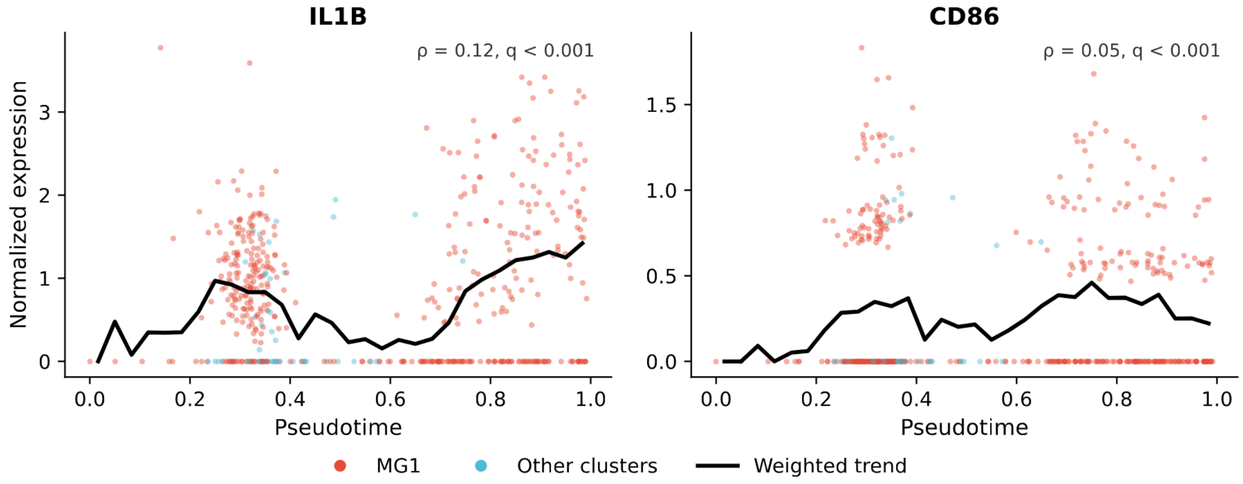

This figure shows the normalized expression of *IL1B* and *CD86* along the pseudotime trajectory for MG0 (**A**) and MG1(**B**) single cell populations. Each dot represents a single cell, colored by gene expression within cluster (red) versus external to the cluster (blue). The black line indicates the weighted expression trend for each gene across pseudotime for MG0 or MG1.

Figure S9. Cross-Species Atlas Comparison Between Human Fetal Mid-Brain and Pigtail Macaque Periventricular White Matter

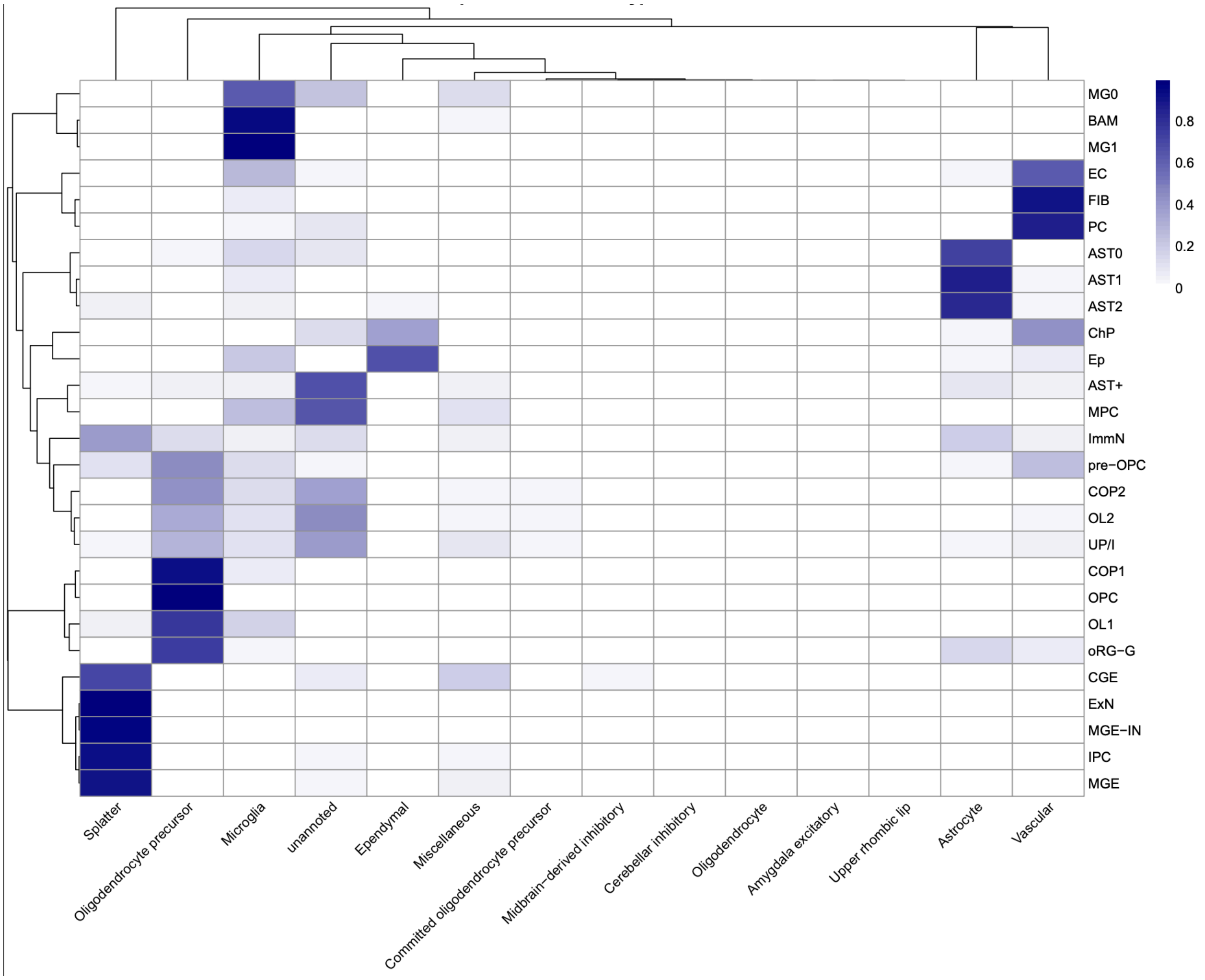

This heatmap represents the proportion of pigtail macaque fetal brain single-cell populations (rows) predicted to correspond to each human midbrain cell type (columns) via Seurat label transfer, with a darker blue indicating a higher proportion of cells assigned to that human cell type. Rows and columns are hierarchically clustered to reveal transcriptomic similarity between macaque and human cell populations. The colored cells represent the percentage of overlap of predicted cell type labels after Seurat label transfer, as a measure of concordance between pigtail macaque and human single cell populations. Abbreviations for single cell populations are shown in the legend of Table S1.
